# Purine Nucleoside Phosphorylase mediated molecular chemotherapy and conventional chemotherapy: A tangible union against chemoresistant cancer

**DOI:** 10.1186/1471-2407-11-368

**Published:** 2011-08-24

**Authors:** Preetinder P Singh, Swapna Joshi, Pamela J Russell, Sham Nair, Aparajita Khatri

**Affiliations:** 1 Oncology Research Centre, Prince of Wales Hospital, Randwick, Sydney, NSW, 2031, Australia; 2 Faculty of Medicine, University of New South Wales, Kensington, NSW, 2036, Australia; 3 Prince Henry's Institute, Block E Level 4, Monash Medical Centre, 246 Clayton Road, Clayton VIC 3168, Australia; 4 Children's Cancer Institute Australia for Medical Research, Lowy Cancer Research Centre, University of New South Wales, Sydney, New South Wales, 2031, Australia; 5 Australian Prostate Cancer Research Centre-Queensland, Princess Alexandra Hospital, Woollangabba, QLD; Cells and Tissue Domain, Institute of Health and Biomedical Innovation, Queensland University of Technology, 60 Musk Avenue Kelvin Grove QLD 4059 Australia; 6 School of Biological Sciences, Macquarie University, Herring Road, North Ryde, Sydney, Australia

**Keywords:** Chemotherapy, Molecular chemotherapy, Purine nucleoside phosphorylase (PNP), Fludarabine Phosphate (Fludara), Gene directed enzyme prodrug therapy (GDEPT), Ovarian cancer, Cancer

## Abstract

**Background:**

Late stage Ovarian Cancer is essentially incurable primarily due to late diagnosis and its inherent heterogeneity. Single agent treatments are inadequate and generally lead to severe side effects at therapeutic doses. It is crucial to develop clinically relevant novel combination regimens involving synergistic modalities that target a wider repertoire of cells and lead to lowered individual doses. Stemming from this premise, this is the first report of *two- and three-way synergies *between Adenovirus-mediated Purine Nucleoside Phosphorylase based gene directed enzyme prodrug therapy (PNP-GDEPT), docetaxel and/or carboplatin in multidrug-resistant ovarian cancer cells.

**Methods:**

The effects of PNP-GDEPT on different cellular processes were determined using Shotgun Proteomics analyses. The *in vitro *cell growth inhibition in differentially treated drug resistant human ovarian cancer cell lines was established using a cell-viability assay. The extent of synergy, additivity, or antagonism between treatments was evaluated using CalcuSyn statistical analyses. The involvement of apoptosis and implicated proteins in effects of different treatments was established using flow cytometry based detection of M30 (an early marker of apoptosis), cell cycle analyses and finally western blot based analyses.

**Results:**

Efficacy of the trimodal treatment was significantly greater than that achieved with bimodal- or individual treatments with potential for 10-50 fold dose reduction compared to that required for individual treatments. Of note was the marked enhancement in apoptosis that specifically accompanied the combinations that included PNP-GDEPT and accordingly correlated with a shift in the expression of anti- and pro-apoptotic proteins. PNP-GDEPT mediated enhancement of apoptosis was reinforced by cell cycle analyses. Proteomic analyses of PNP-GDEPT treated cells indicated a dowregulation of proteins involved in oncogenesis or cancer drug resistance in treated cells with accompanying upregulation of apoptotic- and tumour- suppressor proteins.

**Conclusion:**

Inclusion of PNP-GDEPT in regular chemotherapy regimens can lead to significant enhancement of the cancer cell susceptibility to the combined treatment. Overall, these data will underpin the development of regimens that can benefit patients with late stage ovarian cancer leading to significantly improved efficacy and increased quality of life.

## 1. **Background**

With the limitations of current monotherapies against the heterogeneity of cancer, the concept of combining new and traditional therapies to increase efficacy and lower side effects is generating significant clinical interest. Cancer targeted molecular chemotherapy, engendered by locally administered Gene Directed Enzyme Prodrug Therapy (GDEPT) provides a potent strategy for treating both local and metastatic disease [1,2]., Combining GDEPT with conventional chemotherapy has the potential to increase treatment efficacy and is highly relevant clinically, given that the patients being enrolled in new trials often present with late stage cancer having failed previous chemotherapy [3]. Such combinations are of particular relevance for ovarian cancer patients, most of whom present with late stage disease. With the current treatment option for these patients of platinum chemotherapy (cisplatin and carboplatin), only 20-30% of patients display 5-year survival [4] due to the development of chemo-resistant disease. Although combining platinum therapy with taxanes (paclitaxel, docetaxel) improves efficacy and survival, the development of the chemo-resistant phenotype remains an issue [5]. Overall, combination treatments that are effective in platinum resistant or platinum sensitive patients are needed. Thus, we have evaluated the prospective synergies between Purine Nucleoside Phosphorylase (PNP) mediated GDEPT and chemotherapeutics, docetaxel and carboplatin in multidrug resistant ovarian cancer cells. PNP-GDEPT uses the *E. coli *enzyme, PNP, that can convert systemically administered, FDA approved pro-drug, Fludarabine Phosphate (Fludara), into active toxic metabolites (2-Fluoroadenine (2FA) [6]. Particular advantages of using PNP-GDEPT include (**1**) its potential to kill both dividing and quiescent cells through 2FA incorporation into RNA and DNA [7,8]; (**2**) the potency of "**local bystander" **cell killing effects due to passive, gap-junction independent diffusion of toxic metabolites to surrounding cells [9]; a strong bystander effect is seen even when only 1 in 100-1000 cells express the PNP transgene [10] and (**3**) PNP-GDEPT mediated regression of non-transduced cancer cells at distant sites, namely, '**distant bystander effect' **in immunocompetent mouse models [11]. Preclinically, activity of PNP-GDEPT against cancer (prostate, ovarian, melanoma, colon carcinoma, hepatocellular carcinoma and human glioma) has been proven [10,12] "(reviewed in [1])" and its advantages over HSV/tk GDEPT have been shown [13,14]. *E. coli *PNP has a different active site and substrate binding features from its mammalian counterpart ensuring its clinical relevance [15]. However, the potential application of PNP-GDEPT for treating ovarian cancer remains relatively unexplored, with only one preclinical study being described [10].

Carboplatin displays similar efficacy to cisplatin but is better tolerated and in combination with paclitaxel has become the standard of care for ovarian cancer patients [16,17]. However, this treatment can result in cumulative neurotoxicity and myelosupression which may limit further treatment of these patients. In this study, an alternative to paclitaxel, docetaxel, was investigated based on 1) its activity against relapsed ovarian cancer [18,19] providing symptomatic and survival benefits to responsive patients [20], 2) lower neuropathy and hypersensitivity [20] and 3) its synergistic interactions with carboplatin in ovarian cancer cells [21]. Though, docetaxel/carboplatin combination displayed good therapeutic outcome with relatively lower neuropathy, toxicities leading to grade 3-4 neutropenia and other neutropenic complications have been described [22,23].

We report here the synergistic activity of Adenovirus (Ad)-mediated PNP-GDEPT and docetaxel and/or carboplatin against platinum resistant ovarian cancer cells *in vitro*. To attain a molecular insight into PNP-GDEPT actions and interactions, the protein profile of treated ovarian cancer cells was generated. The involvement of cellular apoptosis genes/proteins in the cytotoxicity of different treatments was assessed given their reported correlations with therapeutic toxicity [24,25]. We anticipate that these data will form a basis for the development of future combination regimens involving PNP-GDEPT in the clinic.

## 2. Methods

### 2.1 Materials

Docetaxel (Commercial name: Taxotere) (Aventis, Pharmaceuticals Inc, NJ), Carboplatin (Pfizer, NSW, Australia) and Fludarabine Phosphate (Fludara), (Schering-Plough, Germany) were used. Antibodies to Bcl-2, Bik, Bax, Survivin, Caspase-7/9 and PARP were used (source, Additional file 1(Table S1).

### 2.2 Cell lines

Ovarian cancer cell lines were maintained either in Dulbecco's Modified Eagle Medium (DMEM) or Roswell Park Memorial Institute medium-1640 (RPMI) media supplemented with 10% foetal calf serum (FCS), 50 U/mL Penicillin and 50 μg/mL Streptomycin (GIBCO/Invitrogen, VIC, Australia) at 37°C, 5% CO_2_, in a humidified incubator.

### 2.3 Viral vectors

Replication defective Ad vectors expressing either Green Fluorescent Protein (AdGFP) or PNP gene (AdPNP) under the control of cytomegalovirus (CMV) promoter were constructed (PNP or GFP genes are cloned in the E1 region of Ad5 genome), propagated and titrated in accordance with the instructions from the AdEasy™ adenoviral vector kit (Stratagene, TX, USA).

### 2.4 Assessment of gene expression of Ad-vector transduced ovarian cancer cells

Cells (24 well plates) infected with Ad-vectors at different multiplicities of infection (moi, plaque forming units (pfu)/cell) were assessed for gene expression at 48 h post infection (pi). GFP: For AdGFP infected cells, the GFP expression was determined by flow-cytometry using CellQuest™ (Version 3.0) software (Becton Dickinson, San Jose, CA). PNP: That the toxic effects of the PNP gene are only possible in the presence of Fludara has been shown unequivocally (with appropriate controls) in our previous studies and thus, is an acceptable measure of PNP activity [11,26]. PNP activity, as determined by the enzymic assay, is accompanied by viral dose dependent ability of the prodrug, Fludara, to kill the transduced cells. The conversion of Fludara to its toxic metabolites by PNP in infected cells was shown by high performance liquid chromatography. Hence in this study, expression of *PNP *(same as used in our previous studies) in AdPNP infected cells was determined by assessment of cell killing in the presence of the prodrug, Fludara, given 48 h post infection using cell viability assays; after 3 to 7 days of incubation with the prodrug a colorimetric assay using the REDOX (4-[3-(4-Iodophenyl)-2-(4-nitrophenyl)-2H-5-tetrazolio]-1, 3-benzene Disulfonate (WST-1) dye (Takara Pty Ltd. Otsu Shiga, Japan) was used. At relevant times, cells were incubated in media containing WST-1 (10: 1) for 2 h and the absorbance measured at 450 nm (Tecan Sunrise, Phoenix Research Products, USA).

### 2.5 Assessment of cytoxicity of docetaxel and/or carboplatin to ovarian cancer cells

Cells treated with different concentrations of docetaxel and/or carboplatin were assessed for cell viability using WST-1 assay at different times (see 2.4).

### 2.6 Evaluation of efficacy of combination of PNP-GDEPT with docetaxel and/or carboplatin

For synergy experiments, cells (plated in triplicate in 96 well plates, also see Additional file 2 (information, A1) were infected with either AdPNP or AdGFP. After 48 h, virus-containing media were replaced with Fludara and/or docetaxel and/or carboplatin containing media. The un-infected cells were treated with docetaxel and/or carboplatin. After 3 -5 days, cell viabilities were assessed by WST-1 assay.

#### 2.6.1 Clonogenic assays to assess cytotoxicity of different treatments

Three days after combination or individual treatments, cells were re-plated in six-well plates. After 6-9 doublings (~2-3 weeks), cell colonies (≥ 50 cells) were stained with crystal violet (0.5% in absolute methanol) and counted.

#### 2.6.2 Evaluation of therapeutic interactions between modalities

The therapeutic interactions between docetaxel, carboplatin and PNP-GDEPT were analysed using the CalcuSyn software (Biosoft, Cambridge, United Kingdom) developed by Chou and Talalay [27,28] that allows statistical evaluation of interactions between 2 or more drugs. This methodology is based on the median effect equation correlating drug and its effects and is used to derive an accurate value of relative potencies of different drugs (e.g. IC_50 _etc). The median effect plot (based on the logarithmic form of Chou's median effect equation) forms the basis of quantification of synergism, summation and antagonism of drug combinations: log (fraction affected/fraction unaffected) vs. log (Dose). A value called Combination Index (CI) is generated that helps quantify the interactions for mutually exclusive and non-exclusive drugs (1983) [27]: A CI < 0.9 implies synergism (> expected additive effect), CI = 0.90-1.10 implies additive and a CI > 1.10 shows antagonism (< expected additive effect) between drugs. Generation of a clinically significant value, 'Dose Reduction Index' (DRI) allows prediction of the fold reduction in individual modality dose when used in combination in comparison to when used alone [29].

### 2.7 Assessment of apoptosis in response to different treatments

Treated or untreated cells were analysed for apoptosis using the M30 CytoDEATH™assay kit (Additional file 1(Table S1) for source), based on binding of an antibody to a Caspase-cleaved epitope of cytokeratin 18-cytoskelatal protein in apoptotic cells and not in viable or necrotic cells (Peviva AB, Bromma, Sweden). Briefly, cells (1 × 10^5^) (24 well plate) given PNP-GDEPT and/or docetaxel and/or carboplatin for 48 h were fixed in methanol (-20°C for 30 minutes), washed twice (PBS with 0.1% Tween 20 (PBST) and incubated with 100 μL of M30 CytoDEATH™ (1:100 dilution) or isotype control IgG_2b _(1:125) antibodies in incubation buffer (PBS with1% bovine serum albumin and 0.1% Tween 20). After a wash in PBST, cells were incubated for 1 h (4°C) with 100 μL of the fluorescein-labelled secondary antibody (FITC 1:70) (Silenus, Melbourne, Australia) and then analysed by flow cytometry (FlowJo Version 7.2.2 (Tree Star, Inc., CA).

### 2.8 Cell-cycle analysis of treated cells

Subconfluent OVCAR-3 cell cultures treated with different treatments were harvested at the indicated time points (0.025% EDTA), washed in ice-cold PBS and fixed using ice-cold ethanol at 4°C for 30 minutes. After two washes in PBS, cellular DNA was stained with propidium iodide solution (50 μg/mL propidium iodide, 0.1 mg/mL RNase A and 0.25% Tween 20 in PBS) for 1 h at 37°C. The percentage of cells in the G_0_/G_1_, S and G_2_/M phases was assessed using flow cytometry.

### 2.9 Protein expression by Western blot analyses

Proteins in cell lysates (50 μg) from variably treated cells were resolved on 10% polyacrylamide gel by SDS-PAGE and then blotted on to nitrocellulose membranes [30]. The blots were incubated overnight in the relevant primary antibody at concentration recommended by the manufacturer (source, Additional file 1 (Table S1) followed by 1 h incubation with secondary antibody (anti-rabbit IgG Horse Radish Peroxidase (HRP) conjugated, 1:1000 or anti-mouse IgG HRP, 1:5000 (source, Additional file 1 (Table S1). The proteins were detected using an enhanced chemiluminescence (ECL) kit (Piercenet, Il, USA); treated membranes were exposed to X-ray film from 1-60 min (as required)-and developed using standard protocols. The protein bands were quantified from these films by densitometry using Quantity One software (Bio-Rad, Hercules, CA).

### 2.10 Analysis of PNP-GDEPT treated samples by shot gun proteomics

Untreated OVCAR-3 cells and those treated with PNP-GDEPT (AdPNP at the moi of 10 pfu/cell for 48 hours and then incubation with Fludara at 1 μg/mL (2.7 μM) were lysed (3 cycles of freeze thawing) and cell lysates (50 μg protein) were resolved by SDS-PAGE using 10% polyacrylamide gel. After Coomassie blue staining (G-250 stain, Bio-Rad, NSW, Australia), from each lane ~ 10-20 gel pieces were excised and peptides extracted (trypsin digest) were processed as described [31] (Mass Spectrometry Unit, UNSW, Sydney, Australia). The digested peptides were separated using HPLC (Ultimate 3000 HPLC and autosampler system, Amsterdam, Netherlands), concentrated and desalted and then subjected to mass spectrometry (MS) (LTQ FT Ultra (Thermo Electron, Bremen, Germany).

#### 2.10.1 Protein identification after mass spectrometry

Peptides and proteins in different samples were identified from extracted tandem mass spectra and peak lists were generated using Mascot Daemon/extract_msn (Matrix Science, London, England, Thermo) using the default parameters submitted to the database search program Mascot (version 2.1, Matrix Science). They were identified through searches on non-redundant NCBI protein database (September 2008, see Additional file 2 (information A2 for details of search parameters and hit criteria). Scaffold (version Scaffold_2.02.01, Proteome Software Inc., Portland, OR) was used to validate MS/MS based peptide and protein identifications (http://www.proteomesoftware.com/index.html). For statistical analyses, datasets (hit values based on scaffold analyses) representing PNP-GDEPT treated and un-treated samples were normalised by square root transformations; log2 transformation was carried out and the data plotted as treated vs. un-treated (data not shown). Subsequently, ratios of log2-transformed data of both samples were obtained to assess up or downregulated proteins. At 95% confidence interval, values of 232.9632 ± 123.8158 were considered significant.

## 3. Results

To explore the potential synergies between PNP-GDEPT, docetaxel and carboplatin, ovarian cancer cell lines representing the most common, adenocarcinoma of epithelial OC, with variable levels of sensitivity to platinum drug treatment were selected (Additional file 3 (Table S2) Based on preliminary evaluations, plating densities that resulted in logarithmic growth at day 7 (maximum duration of our experiments) were used. This avoided potential cell death due to over-confluence/contact-inhibition of control treated cells prior to the termination of our experiments. In a 96 well format, the optimal numbers of cells/well were 7000 (OVCAR-3), 3000 (SKOV-3), 10,000 (A-2780 and 9,000 (Caov-3) (data not shown).

### 3.1 Efficiency of Ad-transduction in different ovarian cancer cell lines

**Figure 1:**
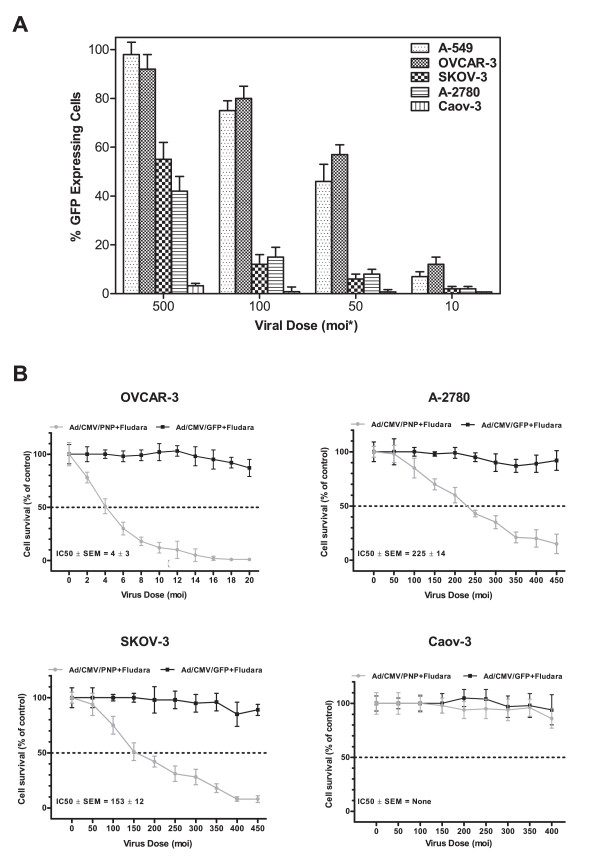
***Panel A***. Evaluation of Ad-transduction in different OC cell lines: Cells infected with AdGFP (10-500 pfu/cell) for 48 h were analysed for GFP expression by flow cytometry. Values represent mean (± SEM) of three experiments. ***Panel B: ***Evaluation of bystander effects associated with PNP-GDEPT in OC cells: Cells infected with AdPNP or AdGFP (control) at different moi followed by the prodrug treatment (Fludara @1 μg/mL(2.7 μM) were evaluated for cell viability (WST-1 assay) on day 5. Graphs show changes in cell viability relative to control (percentage of sham infected control cells) for different cell lines. Values represent mean (± SEM) of three experiments. Corresponding IC_50_ values of PNP-GDEPT in different ovarian cancer cell lines are also shown.

Before evaluating the AdPNP-GDEPT, the permissivity of ovarian cancer cell lines to Ad transductions was evaluated by assessing the efficiency of Ad/CMV/GFP transductions in different cell types at 48 h post infection. This showed a variable level of Ad5-uptake (Figure 1A). At the moi of 100 pfu/cell, OVCAR-3 cells were the most permissive (% GFP expressing cells ± SEM: 80 ± 5) followed by A-2780 (15 ± 4) and SKOV-3 cells (12 ± 4). A highly permissive, lung cancer cell line, A-549, was used as a positive control. Overall, OVCAR-3 cells displayed the highest Ad-permissivity at all Ad/CMV/GFP-doses tested, whilst, Caov-3 cells were almost Ad-refractory with only 3% GFP expressing cells at the high moi of 500 pfu/cell.

### 3.2 Bystander effects of PNP-GDEPT correlate with the efficiency of gene transduction

Evaluation of AdPNP-GDEPT (Fludara: 1 μg/mL (~2.7 μM) in infected cells showed a dose dependent increase in cytotoxicity which correlated with the Ad-permissivity of cell lines (Figure 1B). The toxic effects (% cell death compared to untreated controls ± SEM) were maximal in OVCAR-3 cells (50% ± 5 at moi of 4 pfu/cell) followed by SKOV-3 (25% ± 5 at moi of 100 pfu/cell) and A-2780 (15% ± 7 at moi of 100 pfu/cell) with no effect in Ad-refractory Caov-3 cells. This was also reflected in the IC_50 _values (± SEM) [calculated as moi of AdPNP for 50% cytotoxicity]. Interestingly, the 'bystander effects' also correlated with the Ad-permissivity of cells; in highly permissive OVCAR-3 cells, a significant level of cell inhibition was observed at low viral doses of 1 or 2 moi and up to 90-100% cell growth inhibition was noted when only 12% (at moi of 10, Figure 1A) of cells expressed PNP. Whilst this differed from a previous report [10] that as low as 1% infection was required to kill all cells, the differences may be due to the expression system used, and the use of different cell lines.

### 3.3 Proteins implicated in actions of Ad-PNP GDEPT in ovarian cancer cells

An insight into the molecular changes underlying the cytotoxic effects of PNP-GDEPT may help to understand its interactions with other modalities. Shot-gun proteomics analyses were performed on proteins resolved by SDS-PAGE using cell lysates from untreated and treated (AdPNP at moi of 10 pfu/cell plus 2.7 μM Fludara) platinum-resistant OVCAR-3 cells (see section 2.10). From these, a list of up- and down-regulated proteins was generated (Tables 1 &2); (Note: for description and potential roles of these proteins, see Additional file 4 (Tables S3 and S4). Normalisation of data showed symmetrical distribution, suggesting the similarity of the relative abundance of most proteins in both samples; however, clearly distinctive data points representing potentially unique or differentially abundant proteins were obvious. The stringency of analyses and the low level variation between the two data sets suggested the reliability of these data. Ratio values of treated to untreated cells (range 7925-26529, P < 0.01) indicated clear upregulation of 16 proteins, to levels significantly higher than those predicted for 99% confidence (232.9632 ± 163.636). The abundant expression of *E. coli *PNP, only in PNP-GDEPT treated samples (Scores: 26239 [treated] vs. none [untreated]) provided evidence of its production in Ad/CMV/PNP infected samples. Although, modulated expression of several proteins was seen, only those present in only one sample and which may have a potential role in cancer progression or apoptosis, are listed.

**Table 1:** List of proteins that displayed significantly reduced expression levels in PNP-GDEPT treated samples

Name of the Protein Identified	Hit Value (untreated/treated)	Ratio Transformed log (untreated:treated)^1^
Keratin 5	13/0	19,306
Keratin 77	12/0	18,502
Cadherin 6 (K-cadherin)	7/0	14,999
Desmoplakin	6/0	14,037
Plakoglobin	3/0	10,000
Spondin 1	3/0	10,000
Dynactin 1	2/0	7,925
Agrin	2/0	7,925
Filaggrin	2/0	7,925
Karyopherin alpha1	2/0	7,925
Antiquitin (ALDH7A1)	2/0	7,925
Epoxide hydrolase	2/0	7,925
Insulysin	2/0	7,925
BRI3 binding protein (Cervical cancer 1 proto-oncogene-binding protein KG19)	3/0	10,000
Ribosomal protein L4	5/0	12,924
Eukaryotic translation initiation factor 3 subunit 2 (eIF-3 beta)	3/0	10,000
Mitochondrial trifunctional protein, beta subunit	3/0	10,000
Polymerase (RNA) II (DNA directed) polypeptide E	3/0	10,000
Dihydrolipoamide S-succinyltransferase (E2 component of 2-oxo-glutarate complex)	5/0	12,924
v-ral simian leukemia viral oncogene homolog B (ras related; GTP binding protein) (RalB)	2/0	7,925

^**1**^**Confidence intervals for estimated mean of population**: For 0.95 CI: 232.9632 ± 123.8158; For 0.99 CI: 232.9632 ± 163.636

**Table 2:** List of proteins that displayed significantly increased expression levels in PNP-GDEPT treated samples

Name of the Protein Identified	Hit Value (untreated/treated)	Ratio Transformed log (untreated:treated)^1^
Purine nucleoside phosphorylase *[E. coli]*	0/37	26,239
Poly (ADP-ribose) polymerase (PARP)	0/4	11,609
Progesterone receptor membrane component	0/3	10,000
Angiotensinogen precursor (Serpin A8)	0/3	10,000
Rab13	0/3	10,000
DEAD Box polypeptide	0/2	7,925

^**1**^**Confidence intervals for estimated mean of population**: For 0.95 CI: 232.9632 ± 123.8158; For 0.99 CI: 232.9632 ± 163.636

Overall, PNP-GDEPT treatment lead to general down-regulation of proteins involved in (1) cellular metabolism (lipid, amino acid, carbohydrate and glycolysis) (e.g. polymerase (RNA) II (DNA directed), dihydrolipoamide S-succinyltransferase (E2 component of 2-oxo-glutarate complex), ribosomal protein L4, mitochondrial trifunctional protein), (2) oncogenesis or cancer progression (cadherin, desmoplakin, plakoglobin, karyopherin, spondin, agrin, GTP binding protein and cadherin 6) and (3) drug resistance (antiquitin and epoxide hydrolase). An upregulation of proteins involved in apoptosis, tumour suppression Poly (ADP-ribose) polymerase (PARP) and dead box polypeptide 3) and general DNA synthesis was noted.

### 3.4 Effects of docetaxel or carboplatin treatment on ovarian cancer cell lines

**Figure 2:**
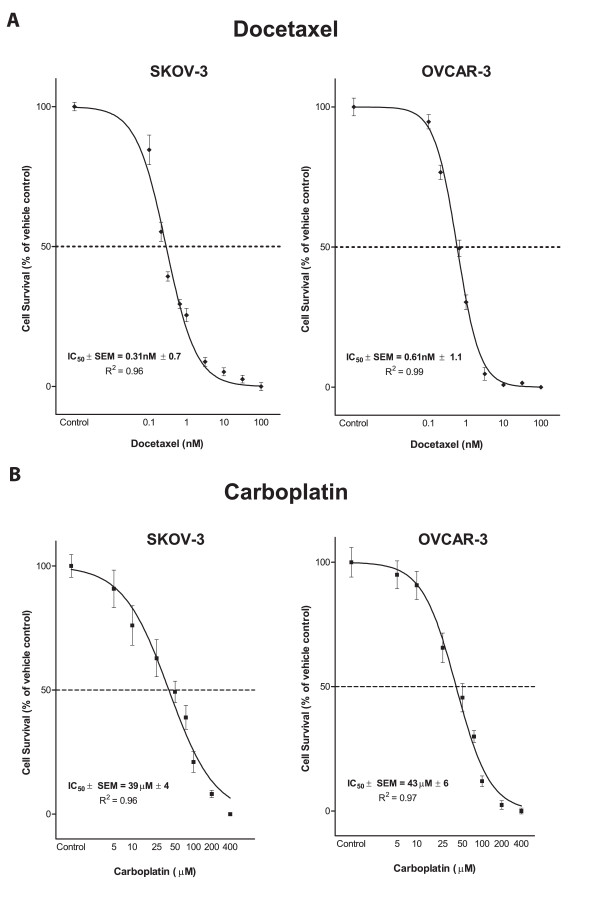
**Response of ovarian cancer cells to docetaxel and carboplatin treatments**: SKOV-3 and OVCAR-3 cells exposed to a range of docetaxel (0.1-316 nM) and carboplatin (1-400 μM) concentrations were evaluated for cell viability using WST-1 assay. Data were plotted as the percentage of vehicle control treated cells (polysorbate 80+ethanol and sterile water, for docetaxel and carboplatin, respectively) using GraphPad Prism, version 5. Dose response curves for SKOV-3 and OVCAR-3 cells treated with docetaxel (panel A) or with carboplatin (panel B) for day 5 are shown. Values represent a mean (± SEM) of three experiments. R2 values > 0.9 suggest that data are reliable and fit the statistical considerations. The corresponding IC_50 _values are also shown.

Growth inhibitory effects of docetaxel against all four ovarian cancer cells lines at different times were variable and dose and time dependent (Figures 2A, and Additional files 5 and 6 (Figure S1 & Table S5). Overall, A-2780 cells were the most sensitive followed by SKOV-3-, OVCAR-3- and Caov-3 cells. SKOV-3 and OVCAR-3 cells were chosen for the following experiments based on their mid-range sensitivity to docetaxel, variable Ad-permissivity and platinum resistance. This allowed us to test the synergies in different scenarios in cisplatin resistant ovarian cancer cells, which would be especially relevant for patients resistant to platinum treatment. The IC_50 _values for docetaxel (Figure 2A) showed SKOV-3 cells (0.31 nM ± 0.7; R^2^:0.96) (Figure 2) to be more docetaxel-sensitive compared to the OVCAR-3 cells (0.61 nM ± 1.1; R^2^:0.99).

Use of cisplatin resistant lines would be particularly relevant to studies involving carboplatin, given its efficacy against cisplatin resistant ovarian cancer [32]. Using a range commonly used in other studies (IC_50 _range ~5 μM-35 μM [33], carboplatin efficacy was found to be dose dependent (Figure 2B). However, only a slight difference in the carboplatin sensitivity (IC_50_) was noted (OVCAR-3: 43.12 μM ± 6; R^2^:0.97 and SKOV-3: 38.73 μM ± 4; R^2^: 0.96) (Figure 2B).

### 3.5 PNP-GDEPT, docetaxel and/or carboplatin act synergistically in ovarian cancer cells *in vitro*

**Figure 3:**
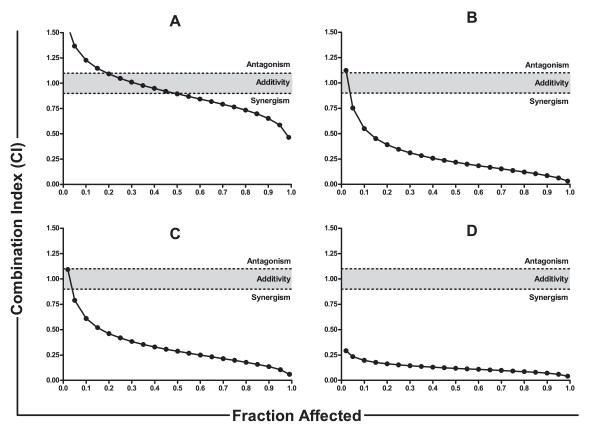
**Analysis of combined drug effects of combination treatments**. Cells treated with different doses of **A**. docetaxel and/or carboplatin, **B**. PNP-GDEPT (Fludara (1 μg/mL (2.7 μM)) and/or docetaxel, **C**. PNP-GDEPT and/or carboplatin D. PNP-GDEPT (Fludara (1 μg/mL (2.7 μM)) and/or docetaxel and/or carboplatin were analysed for cell viability using WST-1 assay on day 5. Graphs representing inhibition of cell growth plotted as a function of increasing doses of docetaxel and/or carboplatin and/or PNP-GDEPT for OVCAR-3 cells are shown in Additional file 7 (Figure A2). Values represent mean (± SEM) of three independent experiments. Based on these, Combination Indices (CIs) for different combinations against the fraction affected (Fa) were plotted to generate CI/Fa plots using *Chou and Talalay's statistical software (CalcuSyn)*. Data for OVCAR-3 cells are shown. Similar data were obtained for SKOV-3 cells. *Synergy is indicated at CI values of < 0.90, Additive interaction is indicated at CI values of 0.90-1.10 and antagonism at CI values of > 1.10*.

**Figure 4:**
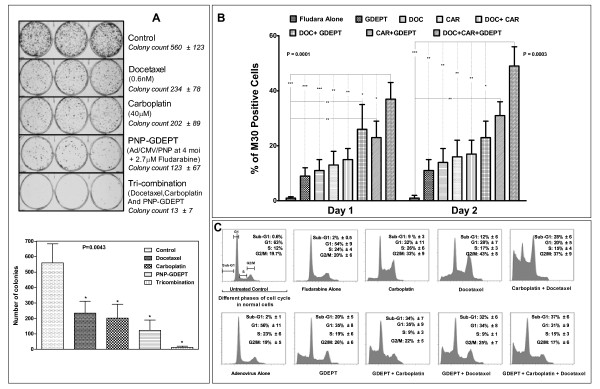
**Effects of different combination treatments on cell survival, apoptosis and cell cycling**: OVCAR-3 or SKOV-3 cells treated with docetaxel, carboplatin and PNP-GDEPT (with Fludara @ 1 μg/mL (2.7 μM)) either alone or in combination were analysed for cell survival by: ***Panel A*. Clonogenic assay, Representative photographs show the crystal violet stained colonies of OVCAR-3 cells given different treatments (docetaxel (0.6 nM), carboplatin (40 μM) and PNP-GDEPT (Ad/CMV/PNP moi of 4 pfu/cell plus 1 μg/mL (2.7 μM) Fludara) either alone or in combination (in triplicate) in a 6 well plate**. The graph and the values in the panel A represents the number of colonies/treatment group and represent mean (± SEM) of two independent experiments. Values were compared by One Way Anova using Dunnet's multiple comparison test. A P value < 0.05 was considered significant (*), however, exact P values for each data set are shown on the graphs. ***Panel B*. Quantitative estimation of apoptosis in ovarian cancer cells (M30 CytoDEATH assay): **SKOV-3 cells were treated with docetaxel (DOC) (3 nM), carboplatin (CAR) (20 μM) and PNP-GDEPT (Ad/CMV/PNP moi of 200 pfu/cell plus 1 μg/mL (2.7 μM) Fludara) either alone or in combination. Cells harvested 1 and 2 days post treatment were immunostained with M30 cytoDEATH antibody followed by flow cytometry. Graph shows the percent of M30 positive cells (represented apoptotic cell death) on different days post treatment with different modalities (alone or in combination). The statistical significance of data on the two days was determined by one-way Anova and using Tukey's multiple comparison tests. Overall P values and the significant differences between control, mono-, and di- and tri-combination treatments effects are displayed: * = P < 1.01, ** = P < 0.001, *** = P < 0.0001. ***Panel C*. Cell cycle analysis**, OVCAR-3 cells treated with different modalities (either alone or in combination; docetaxel (0.6 nM), carboplatin (10 μM) and PNP-GDEPT (Ad/CMV/PNP moi of 10 pfu/cell plus 1 μg/mL (2.7 μM) Fludara) either alone or in combination.) were assessed for cell cycle progression and apoptosis. Cells were harvested 48 h post treatment, RNA was digested and DNA was stained with propidium iodide. The histograms represent the fraction of cells in different phases of the cell cycle after different treatments as determined by flow cytometry. First histogram is representative of different phases of cell cycle in normal untreated cells. While data from one representative experiment is shown, numbers in each panel show the % distribution of cycling cells as mean ± SEM from three independent experiments.

Cell viability (WST-1-based) evaluations of the cells treated with the combinations including docetaxel plus carboplatin, docetaxel plus PNP-GDEPT, carboplatin plus PNP-GDEPT and combination of all three, in general, showed a significant, dose dependent inhibition of cell growth (Additional file 7 (Figure A2) An assessment of interactions between modalities through CalcuSyn analysis [27,28] (see methods for details) of cell viability data generated Combination Index-Fraction affected (CI-Fa) plots. These data showed that at the combination ratio (based on potency) of 1:1, the combined efficacy was additive for the docetaxel/carboplatin combination (Figure 3A and Table 3) (CI:0.89 at IC_50 _dose; R value 0.99), synergistic when PNP-GDEPT was combined with either docetaxel (Figure 3B and Table 3) (CI: 0.21 at IC_50_; R value 0.93) or carboplatin (Figure 3C and Table 3) (CI:0.28 at IC_50_; R value 0.96) and strongly synergistic when all three were combined (Figure 3D and Table 3) (CI:0.11 at IC_50_; R value 0.94). Statistical analyses further revealed the "Dose reduction Index (DRI)" values for each modality in combination to achieve a specific effect for the two cell lines (Additional file 8 (Table S6). In comparison to the individual doses, a dose reduction of up to ~20 fold for PNP-GDEPT, ~27 fold for docetaxel and ~31 fold for carboplatin was predicted to generate 50% cell killing when used in trimodal combination. In addition to evaluation of combinations at the ratio of 1:1, effects were also evaluated at other ratios e.g. 1:2, 2:1, 1:4 (Additional file 9 (Table S7, CI values). Overall, the trends were similar to those obtained when drugs were combined at 1:1 ratio with some variations. To assess whether the drug interactions lead to long term efficacy, clonogenic assays were performed comparing the effects of trimodal therapy with single treatments. Given the fact that carboplatin+docetaxel is the standard treatment for ovarian cancer patients, long term efficacy of the trimodal combination would have direct relevance to the clinic. Treatment of OVCAR-3 cells with, docetaxel (0.6 nM) or carboplatin (40 μM) or PNP-GDEPT (AdPNP at moi of 4 pfu/cell plus 2.7 μM Fludara) alone reduced the number of colonies to ~42%A, 36% and 22% respectively, compared with untreated controls (Figure 4A), whilst the combination of all three reduced colony growth to 2.3%+/-1.3% (13+/-7 cells vs. 560) (Figure 4A). This is slightly greater than an additive effect which would have yielded 3% (0.42 × 0.36 × 0.22 = 0.03) of the controls (17+/-13 cells compared to 560). Within the experimental errors of a clonogenic assay, where it is difficult to accurately assess clonal numbers especially in the untreated cells and due to other factors such as low plating efficiency, colony size and cancer heterogeneity, these results indicate an improvement over additive effects. Thus, though the trends are clear, statistical significance could not be achieved and the final confirmation of the potential of these combinations can only be drawn from exhaustive *in vivo *evaluations. While, these experiments were beyond the scope of this specific study, in another study, we have shown that our *in vitro *findings comparing synergistic responses of prostate cancer cells to PNP-GDEPT with docetaxel (including long term effects as evaluated by clonogenic assays) were supported by the *in vivo *outcomes (Singh et al, *Clin Cancer Res*. 2011, In press).

**Table 3:** Effects of drug interactions between components of different combinations

Modalities	Cell Line	Combination Index (CI^1^)	Drug Alone (×IC_50_)			Dose Reduction Index (×IC_50_)			R^3^	Effect^4^
		**IC^2 ^_50_**	**PNP**	**Doc**	**Car**	**PNP**	**Doc**	**Car**		
Docetaxel Carboplatin	SKOV^5^	0.96	-	5.01	1.34	-	2.7	1.68	0.98	Additive **+**
	OVCAR^6^	0.89	-	2.76	2.16	-	2.1	2.44	0.99	Additive **+**
PNP-GDEPT Docetaxel	SKOV	0.43	8.22	5.01	-	6.12	3.7	-	0.98	Synergy **+++**
	OVCAR	0.21	2.03	2.76	-	7.97	10.82	-	0.93	Strong Synergy **++++**
PNP-GDEPT Carboplatin	SKOV	0.29	8.22	-	1.34	12.36	-	4.7	0.97	Strong Synergy **++++**
	OVCAR	0.28	2.03	-	2.16	5.66	-	9.08	0.96	Strong Synergy ++++
GDEPT Docetaxel Carboplatin	SKOV	0.37	8.22	5.01	1.34	13.96	8.51	5.3	0.96	Strong Synergy **++++**
	OVCAR	0.11	2.03	2.76	2.16	19.89	27.02	31.9	0.94	Very Strong Synergy **+++++**

^1 ^Combination index values generated when drugs were combined at constant ratio of 1:1 e.g. IC_25 _of docetaxel with IC_25 _of carboplatin; ^2^ED, Effective dose, which can result in 50, 75 and 90% of cell killing; ^3^The linear correlation coefficient, R, of the median-effect plot. The acceptable range of 'R' values varies with the type of system used; Enzyme or receptor systems (r > 0.96), tissue culture systems (r > 0.90) and animal experiments (r > 0.85); ^4^Combination effects, CI value 0.90-1.10 signifies additivity (+); CI 0.7-0.85 moderate synergism (++); CI 0.85-0.9 slight synergies (+++); CI 0.30-0.70 synergism (++++); CI 0.10-0.30 strong synergism (+++++) and CI 0.01-010 very strong synergism (++++++); ^5 ^represents SKOV-3; ^6 ^represents OVCAR-3

### 3.6 Evaluation of apoptosis in ovarian cancer cells after different treatments

For these assessments, time points beyond 48 h were not included as then the significant cellular toxicity (apoptosis/cell death) observed with combination regimens could potentially obscure the molecular responses to different treatments [34]. Evaluation of apoptosis in SKOV-3 cells treated with docetaxel (1.5 nM), carboplatin (20 μM) and PNP-GDEPT (AdPNP moi: 150 pfu/cell, 2.7 μMFludara) (doses showing best efficacy) either alone or in combination showed that the numbers of apoptotic cells increased in a time dependent manner in tri-combination treated cells (Figure 4B). The percentage of M30 positive cells (a marker of early apoptosis) was maximal in tri-combination treated cells (49 ± 7) followed by bi-modal (docetaxel/carboplatin (17 ± 5), docetaxel/GDEPT (23 ± 6), carboplatin/GDEPT (31 ± 5), and then individual treatments (PNP-GDEPT (11 ± 4), docetaxel (14 ± 4), carboplatin (16 ± 6). As anticipated, apoptosis achieved in Fludara only treated cells was low (1.5 ± 1), In contrast, when the Fludara was converted by PNP as in cells treated with the combinations including PNP-GDEPT, there was a relatively higher number of apoptotic cells irrespective of the duration of the treatment.

### 3.7 Effects of different treatments on cell cycle in treated cells

Cell cycle analyses of OVCAR-3 cells treated for 48 h with docetaxel (0.6 nM), carboplatin (10 μM) and PNP-GDEPT (AdPNP moi: 10 pfu/cell, 1 μg/mL(2.7 μM) Fludara) either alone or in combination showed a decline in percentage of cells in G1 phase (20-35%) relative to controls, irrespective of the treatments applied (Figure 4C). As expected [35,36] carboplatin (G2/M: 33% ± 9) and docetaxel (G2/M: 43% ± 8) treatments led to an increase in G2/M populations. The percentage of apoptotic cells (sub-G1phase) for different treatments was maximal in tri-combination treated cells (37% ± 6) followed by bi-modal (carboplatin/docetaxel (28% ± 6) < PNP-GDEPT/docetaxel (32% ± 6) < PNP-GDEPT/carboplatin (34% ± 7) and then least in single agent treated cells (carboplatin (9% ± 3) < docetaxel (12% ± 6) < PNP-GDEPT (20% ± 5) with negligible apoptosis in control treated cells. As with M30 analysis, the percentage apoptosis was greater when treatments included PNP-GDEPT. Results were statistically significant when combination treatment was compared with single treatment or control treatment (P ≤ 0.05).

### 3.8 Treatment related effects on pro- and anti-apoptotic proteins

**Figure 5:**
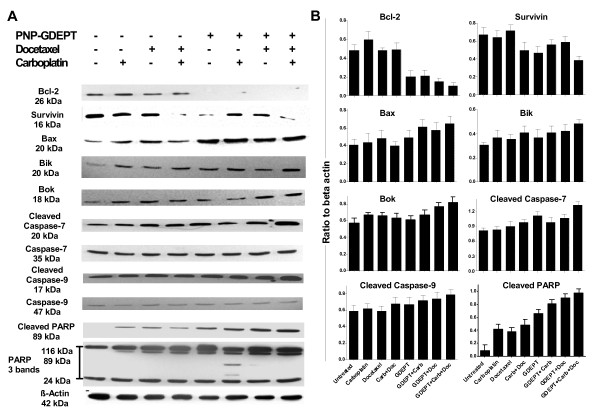
**Evaluation of treatment related effects on key proteins involved in cellular apoptotic pathways in OVCAR-3 cells**: Cell lysates (50 μg) from treated and untreated cells were analysed by SDS-page/western blotting using the appropriate antibodies (panel A). The protein bands were quantified by densitometry relative to β-actin (loading control, representative image in the lowest panel) and expressed as the ratio of specific protein to beta-actin for different treatments (mean ± SEM, n = 2 for two separate experiments) (panel B). Note: only the representative beta-actin image is shown in panel A. For each protein tested, beta actin controls were included separately in the gel (not shown in panel A) and the ratios calculated individually (Panel B).

Mutations in pathways involved in apoptosis modulate their responses to therapy (e.g. chemoresistance) [reviewed in [37]. Apoptosis involves initiation (apoptotic stimulus, e.g., chemotherapeutic or biological agent), effector and execution phases (which decide the fate of the cell) [38]. Thus, relative expressions of protein members representing effector phase [Bcl-2, Bik, Bax, Inhibitor of apoptosis (survivin) and execution phase (caspase-7/9, PARP) were evaluated in the treated cells (Figure 5A &5B) (See Additional file 10 (Table S8) for their correlation with ovarian cancer outcomes). A significant but variable down-regulation of anti-apoptotic genes/proteins (Bcl-2) and up-regulation of pro-apoptotic genes (Bax, Bik and Bok) was achieved when modalities were combined, especially when PNP-GDEPT was included (Figure 5, Additional file 11 (Table S9). Their expression was relatively little affected by either carboplatin or docetaxel used alone or together. Survivin was down-regulated by all combination treatments. Evaluation of PARP (cleaved and un-cleaved) expression in differentially treated cells showed the upregulation of un-cleaved (especially 116 kDa band) and cleaved PARP in response to all treatments; this was even more in the case with combination treatments, and highest when PNP-GDEPT was involved. As Caspase-mediated proteolysis of PARP is a biochemical marker of apoptosis that denotes the final stages of apoptosis leading to DNA fragmentation [39], this indicates that cell killing was occurring in the treated cells.

## 4. Discussion

This study is the first demonstration of potential synergistic anti-tumour interactions between PNP-GDEPT based molecular chemotherapy with docetaxel and carboplatin in cisplatin-resistant ovarian cancer cells. The data are significant given that the ovarian cancer cell lines tested represented subtypes of the epithelial adenocarcinomas encountered in over 80% of patients. Synergy evaluations in complex biological systems such as cells grown in culture can be affected by biological variability, desired dose levels, experimental conditions, such as, temperature, oxygen tension, pH and finally whether synergy is treatment schedule dependent or combination ratio dependent [40]. To address the differences between measurement units of PNP-GDEPT and chemotherapeutic drugs, the combination ratios were based on the potency of the individual components (i.e. doses to achieve a particular effect e.g. IC_50_). We minimised the variability and error in data interpretation by evaluating IC_50 _values in each experiment for individual modalities and evaluating potential synergy using the well-recognized *Chou and Talalay'*s statistical analyses (CalcuSyn software). For simplicity, mutual exclusivity of the component drugs was assumed, as recommended when more than two drugs are involved [27,40].

The efficiency of docetaxel and carboplatin against the chosen ovarian cancer cell lines correlated with preclinical and clinical reports [21,33]. The use of PNP-GDEPT to treat ovarian cancer has been reported in only one study; this was against SKOV-3 cells/tumours, although, the prodrug, 6-methylpurine-2'-deoxyriboside (MePdR), which is yet to be FDA approved, was used [10]. Our *in vitro *data correlated with their outcomes. The use of FDA approved Fludara in our study yields a greater clinical relevance to our findings.

A synergy between modalities means greater efficacy than the two added together. Synergy between PNP-GDEPT and docetaxel and/or carboplatin has important clinical implications; in particular, the ability to use lower doses of each modality should translate in the clinic to decreased side effects and an improved quality of life. An estimation of clinically relevant Dose Reduction Indices made using the CalcuSyn software (Additional file 8 (Table S6) indicated that significant lowering of individual doses when used in combination is possible. This is especially relevant when PNP-GDEPT is given to patients who have had prior- or are undergoing chemotherapy with docetaxel and/or carboplatin treatment. Dose reduction of docetaxel (ranging from ~4-45 fold and carboplatin (from 3-56 fold) was predicted in the trimodal combination with PNP-GDEPT in the multidrug resistant ovarian cancer cells under study. Given the toxicities associated with high dosing of carboplatin or docetaxel alone and in combination [41,42], these are valuable outcomes especially, for treating patients with drug refractory cancer. A decrease in therapeutic doses of PNP-GDEPT could also lower the total Fludara required; this is important given its potential immunosuppressive effects. The data obtained in this study warrant further confirmation *in vivo*. However, we have previously ratified the *in vitro *synergy found between PNP-GDEPT and docetaxel treatment against prostate cancer cells by *in vivo *studies, where a decrease in tumour load both in the prostate and at distant sites in immunocompetent mice was achieved [Singh P et al, 2011, In press, *Clin Can Res*]. Although, synergies were undeniably proven using combinations in multiple ratios, an assessment of sequential administration of different treatments was not done. Further exploration of treatments given in tandem may lead to better synergies; it has been indicated that a tumour containing both wild-type p53 cells as well as p53 mutants could be treated with platinum followed by a taxane. In such a tumour, platinum would first eradicate the wild-type p53 cells after which the taxane would kill those with mutant p53 [43].

Synergies between AdPNP-GDEPT and docetaxel could be partly explained by mutual enhancement stimulated by Ad transduction and docetaxel [44,45]. However, interactions between PNP/Fludara and docetaxel or those between carboplatin and AdPNP-GDEPT are not yet fully understood. Overall, it appears as though the levels of synergies vary between cell types and that trimodal therapy may not be as beneficial for some cell types as expected (e.g. in SKOV-3 cells, bimodal + PNP-GDEPT was as effective as trimodal treatment). A better understanding of these interactions will help in the design of new regimens in cohorts who have undergone pre-existing treatment.

In an effort to understand these interactions, protein studies were performed. Apoptosis was shown to play a significant role in cell death triggered by combining two or three modalities (Figure 5). The most effective apoptotic stimulus (M30 positive cells, sub G1 phase) occurred when bimodal combinations involved PNP-GDEPT or tri-combination treatment was given, accordingly reflected in the cell killing observed in our studies. Interference with cell cycle through processes like DNA damage/microtubule-stabilisation initiates apoptosis. Both carboplatin and docetaxel led to an accumulation of cells in G2/M as shown previously [35,36,46]; combining them led to increased apoptosis (sub G0/G1 phase) suggesting irreversible DNA damage. This may explain the success of carboplatin/taxane combinations in the clinic although, long-term data on the development of the chemoresistant phenotype of ovarian cancers is as yet inadequate [22,47]. Increasing involvement of pro-/anti-apoptotic proteins and caspases which generally regulate the effector phase of apoptosis was observed with multimodal treatments in accordance with the corresponding cell killing synergies. Overall, pro-apoptotic proteins Bax, Bik, Bok, Cleaved Caspase-7 & -9 were up regulated and anti-apoptotic, Bcl-2 and Survivin were down regulated when modalities were combined, albeit to variable levels. A strong protein expression of both caspase-7 and -9 in responses to combination therapies suggests that pathways involving these Caspases (initiated through release of cytochrome c from mitochondria) involving the Bcl-2 family of pro- and anti-apoptotic proteins may be more active in these synergies.

This is the first study to identify protein changes in response to PNP-GDEPT as obtained in OVCAR-3 cells using Shotgun Proteomics [47,48]. That PNP-GDEPT may be acting through involvement in numerous processes ultimately leading to shutdown of cell metabolism, downregulation of some key oncogenes (e.g. cadherin, desmoplakin, plakoglobin, karyopherin, spondin, agrin) and genes involved in drug detoxification (e.g. antiquitin, epoxide hydrolase) with final upregulation of apoptosis (e.g. PARP) or tumour suppressor proteins (e.g. Dead box polypeptide 3) is suggestive of pro-apoptotic effects (Table 3 and Additional file 4 (Tables S3 & S4). Of interest was the downregulation of genes involved in detoxification or drug resistance, which may account for the increased sensitivity of drug resistant OVCAR-3 cells to docetaxel and carboplatin and for enhanced apoptosis observed when PNP-GDEPT was included in combination regimens. This is predictive of its promise for synergies with chemotherapy in the clinic.

Another observation of note is the downregulation of proteins representing "a desmosome model of carcinogenesis" (including desmoplakin, cadherins, plakoglobin, filaggerin) proposed by Chidgey *et al *[48], in which upregulation of c-myc or Bcl-2 promotes uncontrolled cell growth. This was supported by the Bcl-2 downregulation associated with PNP-GDEPT treatment (Figure 5) in our study. Hence, downregulation of this pathway may be one of the major features of PNP-GDEPT efficacy. There was also a possible stress response, with upregulation of proteins involved in purine and pyrimidine metabolism coupled with the downregulation of metabolic pathways. This upregulation of DNA synthesis proteins could also be Ad-transduction mediated, as this is known to trigger the cells into synthesis phase.

## 5. Conclusion

In conclusion, the data indicate that the apoptosis induced by the three way synergy between docetaxel, carboplatin and PNP-GDEPT involves the effector phase mediated by the Bcl-2 family of proteins (pro- and anti-apoptotic) and execution phase involving cleaved Caspase 9, Caspase 7 and PARP. Overall, there is a strong indication that involvement of PNP-GDEPT correlates with a more active involvement of pathways involving downregulation of Bcl-2, survivin and drug resistance proteins, leading to a high apoptotic index as achieved in the synergies involving PNP-GDEPT. These data strongly support the use of PNP-GDEPT in synergistic treatments in the clinic for ovarian cancers that show drug resistance to first line therapies. We anticipate that the information generated in this study will have potential applications against other types of cancers.

## 6. Conflict of interest statement

The authors declare that they have no competing interests.

## 7. Authors' contributions

PS is responsible for the completion of all the experimental work. He was involved in the design and development of this study (and in conducting experiments. He contributed significantly to drafting and writing of this manuscript. SJ was responsible for the western blotting/densitometry studies, and participated in general, in all aspects of this study (experimental planning and conducting) and helped with literature searches, experimental design and data interpretation. PJR provided the overall infrastructural, financial and intellectual support for this study. She was actively involved in the conception, design and development of this work and contributed towards writing and editing of the manuscript. SN provided resources and intellectual input for design and data interpretation of the shotgun proteomic analyses. He was involved in the writing/editing of this manuscript and provided the critical feedback for shotgun proteomics analyses. AK is responsible for the conception, design and development of this study. In addition to coordinating the research, she contributed significantly towards data interpretation and final analyses. She also contributed significantly to literature searches, drafting and writing of this manuscript. All authors have read and approved the manuscript in its final form.

## Pre-publication history

The pre-publication history for this paper can be accessed here:

http://www.biomedcentral.com/1471-2407/11/368/prepub

## Supplementary Material

Additional file 1 **Table S1**. List of primary and secondary antibodies, their respective dilutions and suppliers. Click here for file

Additional file 2 **Additional information S1 and S2**. S1: TEMPLATE FOR SYNERGY EVALUATIONS; S2: SHOT GUN PROTEOMICS PROTOCOLS. Click here for file

Additional file 3 **Table S2**. Properties of OC cell lines used in this study. Click here for file

Additional file 4 **Tables S3 & S4**. Table S3: List of proteins significantly down-regulated in PNP-GDEPT treated samples compared to untreated control cells and their role in cancer. Table S4: List of proteins significantly up regulated in PNP-GDEPT treated samples compared to untreated control cells and their role in cancer. Click here for file

Additional file 5 **Figure S1**. Response of ovarian cancer cells to docetaxel treatment: Four ovarian cancer cell lines were exposed to a range of docetaxel concentrations (0.1-316 nM). WST-1 assay was performed to analyse cell viability on days 2, 3, 4 and 5. Cell viability was plotted as the percentage of vehicle control cells (cells treated with corresponding concentrations of polysorbate 80+ethanol) using GraphPad Prism. Dose response curves for A-2780, SKOV-3, OVCAR-3 and Caov-3 as generated on days 2 (black line), 3 (red line), 4 (purple line) and 5 (brown line) are shown. Click here for file

Additional file 6 **Table S5**. Docetaxel (nM) needed to kill 50% of ovarian cancer cells (IC_50_). Click here for file

Additional file 7 **Figure S2**. Evaluation of cell growth inhibitory effects of different combinations in OVCAR-3 cells: Cells treated with different doses of A. docetaxel and/or carboplatin, B. PNP-GDEPT (Fludara (1 μg/mL, 2.7 μM) and/or docetaxel, C. PNP-GDEPT and/or carboplatin D. PNP-GDEPT (Fludara (1 μg /mL, 2.7 μM) and/or docetaxel and/or carboplatin were analysed for cell viability using WST-1 assay on day 5. Graphs representing inhibition of cell growth plotted as a function of increasing doses of docetaxel and/or carboplatin and/or PNP-GDEPT for OVCAR-3 cells are shown. *Some combinations led to slightly greater cell growth inhibition compared with either alone*. Values represent mean (± SEM) of three independent experiments. The P values on the graph indicate the significance of trends at various time points calculated using Two Way Anova analyses. Click here for file

Additional file 8 **Table S6**. Dose Reduction Index (DRI) values for different modalities when used in combination in platinum resistant ovarian cancer cells. Click here for file

Additional file 9 **Table S7**. Interactions between different components of the combination treatments at different drug combination ratios in OC cells. Click here for file

Additional file 10 **Table S8**. Genes/Proteins expressions and their correlation with OC outcomes. Click here for file

Additional file 11 **Table S9**. Summary of treatment related effects on different pro and anti-apoptotic proteins. Click here for file
