# Supplementary material for: Purine Nucleoside Phosphorylase mediated molecular chemotherapy and conventional chemotherapy: A tangible union against chemoresistant cancer
Source: BMC Cancer. 2011 Aug 24;11:368. doi: 10.1186/1471-2407-11-368 (PMC3185280; doi:10.1186/1471-2407-11-368)
Supplement: Additional file 1 — Table S1. List of primary and secondary antibodies, their respective dilutions and suppliers. [file 1471-2407-11-368-S1.DOC]

**Additional File 1**

**Title: Table S1**

**Description: List of primary and secondary antibodies, their respective dilutions and suppliers**

| **Antibodies** | **Antigen** | **Source** | **Class/Reactivity** | **Dilutions used** | **Supplier** |
| --- | --- | --- | --- | --- | --- |
| **Anti-Bcl -2 (B cell leukaemia-** 2**) protein family (Effector pathway)** | BCL-2 (50E3)  (anti-apoptotic) | Rabbit | Monoclonal IgG  H, M, R, Mk | 1:1000 (WB)^1^ | Cell Signalling Technology, USA |
|  | Bax (B-9): sc-7480  (pro-apoptotic) | Mouse | Monoclonal IgG  H, M, R | 1:100 (WB) |  |
|  | Bik  (pro-apoptotic) | Rabbit | Monoclonal IgG  H, M, R, Mk | 1:800 (WB) |  |
|  | Bok (pro-apoptotic) | Rabbit | Monoclonal IgG  H, M, Mk | 1:800 (WB) |  |
| **Anti-apoptosis proteins (execution pathway)**  **Apoptosis sampler kit** | Caspase-3 | Rabbit | Monoclonal IgG  H, M, R | 1:800 (WB) |  |
|  | Cleaved Caspase-3 | Rabbit | Monoclonal IgG  H, M, R, B | 1:800 (WB) |  |
|  | Caspase-7 | Rabbit | Monoclonal IgG  H, M, R | 1:800 (WB) |  |
|  | Cleaved Caspase-7 | Rabbit | Monoclonal IgG  H, M, R | 1:800 (WB) |  |
|  | Caspase-9 | Rabbit | Monoclonal IgG  H only | 1:800 (WB) |  |
|  | Cleaved Caspase-9 | Rabbit | Monoclonal IgG  H only | 1:800 (WB) |  |
|  | PARP and cleaved PARP | Rabit | Monoclonal IgG  H, M only | 1:800 (WB) |  |
| **Anti-Inhibitors of Apoptosis**  **(Effector pathway)** | Survivin (71G4) | Rabbit | Monoclonal IgG  H, M, R | 1:1000 (WB) |  |
| **Anti- Cytokeratin 18** | M30-CytoDeath | Rabbit | Monoclonal IgG  H, M, R | 1:50 (FC)^2^ | Alexis, Peviva, Sweden |
| **Secondary antibodies** |  | Rabbit | Rabbit anti-mouse | 1:5000 (FC) | Cell Signalling Technology, USA |
|  |  | Goat | Goat anti-rabbit | 1:1000 (FC) |  |

^1^Western Blotting; ^2^ Flow Cytometry
