# Supplementary material for: Purine Nucleoside Phosphorylase mediated molecular chemotherapy and conventional chemotherapy: A tangible union against chemoresistant cancer
Source: BMC Cancer. 2011 Aug 24;11:368. doi: 10.1186/1471-2407-11-368 (PMC3185280; doi:10.1186/1471-2407-11-368)
Supplement: Additional file 2 — Additional information S1 and S2. S1: TEMPLATE FOR SYNERGY EVALUATIONS; S2: SHOT GUN PROTEOMICS PROTOCOLS. [file 1471-2407-11-368-S2.DOC]

**Additional File 2**

**Title: Addiotinal information S1 and S2**

**Description: A1: TEMPLATE FOR SYNERGY EVALUATIONS; A2: SHOT GUN PROTEOMICS PROTOCOLS**

| TREATMENT II  (XIC50) | TREATMENT I (XIC50) | | | | | |
| --- | --- | --- | --- | --- | --- | --- |
|  | Control | ⅛IC_50_ | ¼IC_50_ | ⅓IC_50_ | ½IC_50_ | IC_50_ |
|  | ⅛IC_50_ |  |  |  |  |  |
|  | ¼IC_50_ |  |  |  |  |  |
|  | ⅓IC_50_ |  |  |  |  |  |
|  | ½IC_50_ |  |  |  |  |  |
|  | IC_50_ |  |  |  |  |  |

**S1: TEMPLATE FOR SYNERGY EVALUATIONS**

**S2: SHOT GUN PROTEOMICS PROTOCOLS**

**Mass Spectrometry**

Untreated cells treated and those treated with PNP-GDEPT were analyzed for protein profiles using shotgun proteomics approach [1-3]. Cell lysates (50 µg) were electrophoresed on 10% polyacrylamide gel as described (*section 2.1.2.3*). Immediately after Coomassie staining, gel (stored in water at RT) was taken for mass spectrometry analyses to Mass Spectrometry Unit, BMSF at UNSW*.* Gel bands were incubated with NH_4_HCO_3_/CH_3_CN (10 mM, 1:1, 200 µl) until clear (~2-4 hr) then CH_3_CN (2 x 50 µl, 10 min) and dried under vacuum (SpeedVac, Savant, Farmingdale, NY). Trypsin (~100 ng) in NH_4_HCO_3_ (10mM, 25 µl) was added and the solution left at 37°C for 14 h. The gel pieces were washed with H_2_O (0.1% formic acid) (50 µl) and H_2_O: CH3 CN (1:1) (0.1% formic acid) (50 µl) for 10 min and the combined extracts dried and peptides dissolved in H_2_O (0.1% HFBA, 20 µl) [4].

Digest peptides were separated by nano-LC using an Ultimate 3000 HPLC and autosampler system (Dionex, Amsterdam, Netherlands). Samples (2.5 µl) were concentrated and desalted onto a micro C18 precolumn (500 µm x 2 mm, Michrom Bioresources, Auburn, CA) with H_2_O: CH_3_ CN (98:2, 0.05 % HFBA) at 20 µl/min. After a 4 min wash the pre-column was switched (Valco 10 port valve, Dionex) into line with a fritless nano column (75µ x ~10cm) containing C18 media (5 µ, 200 Å Magic, Michrom) manufactured according to Gatlin [5]. Peptides were eluted using a linear gradient of H_2_O: CH_3_ CN (98:2, 0.1 % formic acid) to H_2_O: CH_3_ CN (64:36, 0.1 % formic acid) at 350 nl/min over 30 min. High voltage 1800 V) was applied to low volume tee (Upchurch Scientific) and the column tip positioned ~ 0.5 cm from the heated capillary (T=200°C) of a LTQ FT Ultra (Thermo Electron, Bremen, Germany) mass spectrometer. Positive ions were generated by electrospray and the LTQ FT Ultra operated in data dependent acquisition mode (DDA).

A survey scan m/z 350-1750 was acquired in the FT ICR cell (Resolution = 100,000 at m/z 400, with an initial accumulation target value of 1,000,000 ions in the linear ion trap). Up to the 7 most abundant ions (>2500 counts) with charge states of +2 or +3 were sequentially isolated and fragmented within the linear ion trap using collisionally induced dissociation with an activation q = 0.25 and activation time of 30 ms at a target value of 30,000 ions. M/z ratios selected for MS/ MS were dynamically excluded for 30 seconds.

Peak lists were generated using Mascot Daemon/extract_msn (Matrix Science, London, England, Thermo) using the default parameters, and submitted to the database search program Mascot (version 2.1, Matrix Science). Search parameters were: Precursor tolerance 4 ppm and product ion tolerances ± 0.6 Da; Met (O) and Cys-carboxyamidomethylation specified as variable modification, enzyme specificity was trypsin, 1 missed cleavage was possible and the non-redundant NCBI protein database (September 2008) was searched.

**Protein identification after mass spectrometry**

Scaffold (version Scaffold_2.02.01, Proteome Software Inc., Portland, OR) was used to validate MS/MS based peptide and protein identifications (<http://www.proteomesoftware.com/index.html>). Scaffold peptide identifications were performed based on the Peptide Prophet algorithm [6]. Subsequently, using the Scaffold software analyses, different data-sets were compared. For these, peptide identifications were accepted if they could be established at greater than 95% probability as specified by the Peptide Prophet algorithm [6]. Protein identifications were assigned by the Protein Prophet algorithm [7] and were accepted if they could be established at greater than 90% probability and contained at least 2 identified peptides. Proteins that contained similar peptides and could not be differentiated based on MS analysis alone were grouped to satisfy the principles of parsimony which is a novel way of handling the noise (false positives) in the protein interaction data [8].

**Analyses parameters/protocol for Scaffold data:** To check the relevance of scaffold data, statistical analysis was performed as described below. Briefly, ratios of log2-transformed data of both samples were obtained to assess up or downregulated proteins (whole ratio- based dataset was treated as a single population; zero values were changed to 0.0001 to avoid division by zero error). The following are the details of the analysis performed

**Values entered:**

X = log2 transformed ratios

**Summary of results (values):**

Number of proteins (N) = 745

-X = 173557.55299999984

-X2 = 2186160044.0213213

Mean = 232.9632

Variance = 2884042.3768

Std. dev. = 1698.2469

Std. error = 62.219

df = 744

tcrit(.05) = 1.99

tcrit(.01) = 2.63

**Confidence intervals for estimated mean of population:**

For 0.95 CI: 232.9632±123.8158

For 0.99 CI: 232.9632±163.636

At 95% confidence interval, values of 232.9632±123.8158 were considered significant. Due to very little variance in the data from two data sets, there is not much difference at the 99% confidence interval except for the variation in standard error values (Figure 4.5). In the absence of replicates, the statistical significance of the data could not be predicted, however, the stringency of analyses and the very little variation between the two data sets suggested the accuracy and reliability of the data analyses.

**References:**

1. Hu L, Ye M, Jiang X, Feng SZou H. (2007). Advances in hyphenated analytical techniques for shotgun proteome and peptidome analysis--a review*.* *Anal Chim Acta*;**598:** 193-204.

2. Marcotte EM. (2007). How do shotgun proteomics algorithms identify proteins? *Nat Biotechnol*;**25:** 755-7.

3. Fournier ML, Gilmore JM, Martin-Brown SAWashburn MP. (2007). Multidimensional separations-based shotgun proteomics*.* *Chem Rev*;**107:** 3654-86.

4. Shevchenko A, Wilm M, Vorm OMann M. (1996). Mass spectrometric sequencing of proteins silver-stained polyacrylamide gels*.* *Anal Chem*;**68:** 850-8.

5. Gatlin CL, Kleemann GR, Hays LG, Link AJYates JR, 3rd. (1998). Protein identification at the low femtomole level from silver-stained gels using a new fritless electrospray interface for liquid chromatography-microspray and nanospray mass spectrometry*.* *Anal Biochem*;**263:** 93-101.

6. Keller A, Nesvizhskii AI, Kolker EAebersold R. (2002). Empirical statistical model to estimate the accuracy of peptide identifications made by MS/MS and database search*.* *Anal Chem*;**74:** 5383-92.

7. Nesvizhskii AI, Keller A, Kolker EAebersold R. (2003). A statistical model for identifying proteins by tandem mass spectrometry*.* *Anal Chem*;**75:** 4646-58.

8. Guimaraes KS, Jothi R, Zotenko EPrzytycka TM. (2006). Predicting domain-domain interactions using a parsimony approach*.* *Genome Biol*;**7:** R104.
