# Supplementary material for: Purine Nucleoside Phosphorylase mediated molecular chemotherapy and conventional chemotherapy: A tangible union against chemoresistant cancer
Source: BMC Cancer. 2011 Aug 24;11:368. doi: 10.1186/1471-2407-11-368 (PMC3185280; doi:10.1186/1471-2407-11-368)
Supplement: Additional file 3 — Table S2. Properties of OC cell lines used in this study. [file 1471-2407-11-368-S3.DOC]

**Additional File 3**

**Title: Table A2**

**Description: Properties of OC cell lines used in this study.**

**Table S2: Properties of OC cell lines used in this study.**

| **Cell Line*** | **Features** | **References** |
| --- | --- | --- |
| **SKOV-3** | Cystadenocarcinoma; cells are resistant to tumor necrosis factor and to several cytotoxic drugs including diphtheria toxin, cis-platinum and Adriamycin; high expression of Her-2 neu receptor; often used as a positive control in Her-2 neu target based strategies; p53 null | [1, 2] |
| **OVCAR-3** | Cystadenocarcinoma; derived from malignant ascites of a patient with progressive adenocarcinoma resistant to cisplatin therapy; resistant to clinically relevant concentrations of adriamycin, melphalan and cisplatin; both cultured cells and xenografts exhibit androgen and oestrogen receptors; often used in studies looking at mechanisms of platinum drug resistance and presence of hormone receptor is useful to evaluate hormonal therapy; highly permissive to Ad infections; mutations in p53 *(mut R 248 Q)* | [3] |
| **A-2780** | Serous cystadenocarcinoma; derived from a patient never treated with chemotherapy; very fast growing; wild type p53 | [4] |
| **Caov-3** | Cystadenocarcinoma; cisplatin resistant, minimal expression of integrins and CAR receptor; non-permissive to Ad infection; mutations in p53*(mut Q 136 term)* | [1] |

* Source American Type Culture Collection, U.S.A ([www.atcc.org](http://www.atcc.org/))

References cited:

1. Fogh J, Wright WCLoveless JD. (1977). Absence of HeLa cell contamination in 169 cell lines derived from human tumors*.* *J Natl Cancer Inst*;**58:** 209-14.

2. Fogh J, Fogh JMOrfeo T. (1977). One hundred and twenty-seven cultured human tumor cell lines producing tumors in nude mice*.* *J Natl Cancer Inst*;**59:** 221-6.

3. Hamilton TC, Young RC, McKoy WM, Grotzinger KR, Green JA, Chu EW *et al.* (1983). Characterization of a human ovarian carcinoma cell line (NIH:OVCAR-3) with androgen and estrogen receptors*.* *Cancer Res*;**43:** 5379-89.

4. Behrens BC, Hamilton TC, Masuda H, Grotzinger KR, Whang-Peng J, Louie KG *et al.* (1987). Characterization of a cis-diamminedichloroplatinum(II)-resistant human ovarian cancer cell line and its use in evaluation of platinum analogues*.* *Cancer Res*;**47:** 414-8.
