# Supplementary material for: Purine Nucleoside Phosphorylase mediated molecular chemotherapy and conventional chemotherapy: A tangible union against chemoresistant cancer
Source: BMC Cancer. 2011 Aug 24;11:368. doi: 10.1186/1471-2407-11-368 (PMC3185280; doi:10.1186/1471-2407-11-368)
Supplement: Additional file 4 — Tables S3 & S4. Table S3: List of proteins significantly down-regulated in PNP-GDEPT treated samples compared to untreated control cells and their role in cancer. Table S4: List of proteins significantly up regulated in PNP-GDEPT treated samples compared to untreated control cells and their role in cancer. [file 1471-2407-11-368-S4.DOC]

| **Name of the Protein**  **Identified** | **Properties (References)** |
| --- | --- |
|  |  |
| **Keratin 5** | Keratin gene family; less defined role in cancer; connect with desmosomes (desmoplakin) to form extensive cadherin-mediated cytoskeletal architectures [1] |
| **Keratin 77** | Keratin gene family; less defined role in cancer |
| **Cadherin 6** | Cell adhesion molecule that maintains tissue integrity; Also known as K-cadherin; may lead to an aggressive phenotype during carcinogenesis; overexpression showed in OC patient specimens and established cell lines e.g. OVCAR-3; prognostic marker for OC and renal cell carcinoma [2-8] |
| **Desmoplakin** | Cell adhesion protein associated with desmosomes (desmosomes are intercellular junctions that tightly link adjacent cells); a marker for epithelial cancers; progesterone receptor mediated up regulation in breast cancer [9-16] |
| **Plakoglobin** | A component of desmosome; close association with cadherins; also called γ-catenin; an inducer of c-Myc and BCL-2 protein in human squamous carcinoma cells; nuclear accumulation of plakoglobin with concomitant increase in Bcl-2 shown in CRPC [16-18] |
| **Spondin 1** | Extracellular matrix protein (ECM), also known as SPON1; overexpressed in OC and in OVCAR-3 cells ; a potential diagnostic marker for OC; a predictive marker for palliative 5-FU-based chemotherapy in metastatic colorectal cancer [19-22] |
| **Dynactin 1** | Inhibitor of P53 mediated apoptosis: Macromolecular complex consisting of 10-11 subunits ( from 22 to 150 kDa); binds to microtubules and cytoplasmic dynein; involved in a diverse array of cellular functions and cell cycle progression; being a ‘microtubule-associated protein’ serves as a potential target for cancer chemotherapy e.g. taxanes [23-26] |
| **Agrin** | Angiogenesis and cell proliferation; A key component of Heparan sulfate proteoglycans (HSP) that have implications in cancer cell growth, invasion, metastasis, and angiogenesis; associated with the formation of septal blood vessels in liver cirrhosis, and neoangiogenesis in the hepatocellular carcinoma (HCC); a triggering factor for cell proliferation in osteosarcoma; associated with aggressive phenotype of glioblastoma multiforme, an aggressive form of brain cancer [27-32] |

**Additional File 4: Title:Table S3 &S4**

**Description: Table S3: List of proteins significantly down-regulated in PNP-GDEPT treated samples compared to untreated control cells and their role in cancer. Table S4: List of proteins significantly up regulated in PNP-GDEPT treated samples compared to untreated control cells and their role in cancer**

**Table S3: List of proteins significantly down-regulated in PNP-GDEPT treated samples compared to untreated control cells and their role in cancer.**

| **Filaggrin** | Intermediate filament-associated proteins (IFAP) that bind to keratins bundles in epithelial cells; based on their role on cell morphology and nuclear integrity may have a potential role in apoptosis [33-36] |
| --- | --- |
| **Karyopherin alpha1** | Oncogene; Involvement in nucleo-cytoplasmic trafficking and found to be crucial for protein and RNA subcellular localization; a well defined role in nuclear envelope component assembly, mitosis and replication; overexpressed in cervical cancer and is critical for cancer cell survival and proliferation; karyopherin alpha2 expression predicts poor survival in patients with advanced breast cancer; a potential oncogenic role by the activation of PI3-kinase/AKT- pathway [37-40] |
| **Antiquitin (ALDH7A1)** | Detoxification molecule; a key member of aldehyde dehydrogenase (ALDH) gene family; encodes enzyme that cause detoxification of various pharmaceutical compounds via NAD(P)(+)-dependent oxidation; overexpressed in various tumours and have a potential role in resistance to multiple chemotherapeutic drugs [41-43] |
| **Epoxide hydrolase** | Detoxification: Also known as Epoxide hydratase; functions in detoxication during drug metabolism; expressed in several cancers and activity shown in cancer cell lines e.g. SKOV-3, ES-2, A-549, PC-3 and Du-145; potential implication in anti-cancer drug resistance more specifically in hepatocellular carcinoma and prostate cancer [44-46] |
| **Insulysin** | Insulin degrading enzyme (IDE); activity detected in breast and ovarian tumour tissues and cell lines e.g. OVCAR-3 cell line is positive for expression [47-50] |
| **BRI3 binding protein**  **(Cervical cancer 1 proto-oncogene-binding protein KG19)** | Often named as endoplasmic reticulum (ER) resident protein; mediates cell fate by interacting between ER and mitochondria [51, 52] |
| **Ribosomal protein L4** | Protein synthesis; belongs to the L4E family of ribosomal proteins that is a component of the 60S subunit; overexpressed in doxorubicin resistant colon cancver cell lines; overexpression shown in PC cell lines e.g. PC-3 and Du 145; serves as target for anti-cancer therapies that are directed against protein synthesis [53-55] |
| **Eukaryotic translation initiation factor 3 subunit 2 (eIF-3 beta)** | Also known as EIF3S2 or (TGF-beta receptor-interacting protein 1) (TRIP-1) an initiation factor that regulates mRNA translation and cell growth; abnormal expression detected in various cancers; relatively less studied; potential implications in diagnosis, prognosis, and treatment of human cancers [56, 57] |
| **Mitochondrial trifunctional protein, beta subunit** | Regulates mitochondrial beta oxidation pathways; not related to cancer as yet but but as a general fact mitochondrial defects have been related to a variety of cancer [58, 59] |
| **Polymerase (RNA) II (DNA directed) polypeptide E** | RNA synthesis: A multi-subunit complex which mediates ribonucleotide synthesis; role in cancer is not reported, PNP-GDEPT inhibits RNA synthesis |
| **Dihydrolipoamide S-succinyltransferase (E2 component of 2-oxo-glutarate complex)** | A defined role in carbohydrate metabolism (Tri-carboxylic acid cycle) [60] |
| **v-ral simian leukemia viral oncogene homolog B (ras related; GTP binding protein) (RalB)** | Oncogene; RalA and RalB are members of Ras family; RalA is required for tumorigenesis and RalB is important for tumor survival; a definitive role of Ral proteins has also been shown in cancer cell migration and metastatic tumor invasion [61-65] |

**^1^Confidence intervals for estimated mean of population**: For 0.95 CI:232.9632±123.8158; For 0.99 CI: 232.9632±163.636

**Table S4: List of proteins significantly up regulated in PNP-GDEPT treated samples compared to untreated control cells and their role in cancer**

| **Name of the Protein**  **Identified^1^** | **Properties (References)** |
| --- | --- |
|  |  |
| **Purine nucleoside phosphorylase [E. coli]** | Enzyme used in this GDEPT approach [66] |
| **Poly (ADP-ribose) polymerase (PARP)** | PARP is involved in DNA repair in response to some form of stress; can be cleaved by caspase-3 both *in vitro and in vivo*; helps cells to maintain their viability; cleavage of PARP facilitates cellular disassembly; a marker for cells undergoing apoptosis [67-77] |
| **Progesterone receptor membrane component** | Role is controversial if its favours or opposes cancer activities; overexpression shown in several cancers and its related with cancer cell survival: also known as PGRMC; in spite of its name, PGRMC1 is not a progesterone receptor; PGRMC1 plays an important role in promoting OC cell viability and attenuating PGRMC1's action increased OC cell sensitivity to CDDP mediated apoptosis; reported as a biomarker in breast cancer [78-83] |
| **Angiotensinogen precursor (Serpin A8)** | Serpins (SERine Proteinase Inhibitors); functional expression in OC and involved in tumor progression and angiogenesis; involved in the regulation of tumor angiogenesis especially in receptor negative breast cancer [84-86] |
| **Rab13** | Ras family related; Rab13 may play an important role in the assembly of tight junctions and thus in the establishment of polarity in epithelial cells; Detected in several types of epithelia, including intestine, kidney, liver and in endothelial cells; a specific role in cancer is yet to be determined [87, 88] |
| **DEAD Box polypeptide 3** | Tumour suppressor gene; belongs to DEAD box RNA helicase family; inhibits colony formation activity of HCC, cervical carcinoma, colon cancer, and murine fibroblast cells [89] |

**^1^Confidence intervals for estimated mean of population**: For 0.95 CI: 232.9632±123.8158; For 0.99 CI: 232.9632±163.636

**References:**

1. Kouklis PD, Hutton EFuchs E. (1994). Making a connection: direct binding between keratin intermediate filaments and desmosomal proteins*.* *J Cell Biol*;**127:** 1049-60.

2. Sellar GC, Li L, Watt KP, Nelkin BD, Rabiasz GJ, Stronach EA *et al.* (2001). BARX2 induces cadherin 6 expression and is a functional suppressor of ovarian cancer progression*.* *Cancer Res*;**61:** 6977-81.

3. Xiang YY, Tanaka M, Suzuki M, Igarashi H, Kiyokawa E, Naito Y *et al.* (1994). Isolation of complementary DNA encoding K-cadherin, a novel rat cadherin preferentially expressed in fetal kidney and kidney carcinoma*.* *Cancer Res*;**54:** 3034-41.

4. Li G, Passebosc-Faure K, Gentil-Perret A, Lambert C, Genin CTostain J. (2005). Cadherin-6 gene expression in conventional renal cell carcinoma: a useful marker to detect circulating tumor cells*.* *Anticancer Res*;**25:** 377-81.

5. Marshall FF. (2005). The level of cadherin-6 mRNA in peripheral blood is associated with the site of metastasis and with the subsequent occurrence of metastases in renal cell carcinoma*.* *J Urol*;**173:** 1919.

6. Paul R, Necknig U, Busch R, Ewing CM, Hartung RIsaacs WB. (2004). Cadherin-6: a new prognostic marker for renal cell carcinoma*.* *J Urol*;**171:** 97-101.

7. Shimazui T, Yoshikawa K, Uemura H, Hirao Y, Saga SAkaza H. (2004). The level of cadherin-6 mRNA in peripheral blood is associated with the site of metastasis and with the subsequent occurrence of metastases in renal cell carcinoma*.* *Cancer*;**101:** 963-8.

8. Shimazui T, Yoshikawa K, Uemura H, Kawamoto R, Kawai K, Uchida K *et al.* (2003). Detection of cadherin-6 mRNA by nested RT-PCR as a potential marker for circulating cancer cells in renal cell carcinoma*.* *Int J Oncol*;**23:** 1049-54.

9. Ben-Ze'ev A. (1986). Tumor promoter-induced disruption of junctional complexes in cultured epithelial cells is followed by the inhibition of cytokeratin and desmoplakin synthesis*.* *Exp Cell Res*;**164:** 335-52.

10. Dervan PA, Gilmartin LG, Johnston PGCarney DN. (1988). Desmosomal plaque proteins are preserved in all grades of breast cancer. An immunohistochemical study utilizing monoclonal antibodies to desmoplakin*.* *Am J Surg Pathol*;**12:** 855-60.

11. Gallicano GI, Kouklis P, Bauer C, Yin M, Vasioukhin V, Degenstein L *et al.* (1998). Desmoplakin is required early in development for assembly of desmosomes and cytoskeletal linkage*.* *J Cell Biol*;**143:** 2009-22.

12. Green KJ, Stappenbeck TS, Noguchi S, Oyasu RNilles LA. (1991). Desmoplakin expression and distribution in cultured rat bladder epithelial cells of varying tumorigenic potential*.* *Exp Cell Res*;**193:** 134-43.

13. Green KJ, Stappenbeck TS, Parry DAVirata ML. (1992). Structure of desmoplakin and its association with intermediate filaments*.* *J Dermatol*;**19:** 765-9.

14. Jones JC Grelling KA. (1989). Distribution of desmoplakin in normal cultured human keratinocytes and in basal cell carcinoma cells*.* *Cell Motil Cytoskeleton*;**13:** 181-94.

15. Pang H, Rowan BG, Al-Dhaheri MFaber LE. (2004). Epidermal growth factor suppresses induction by progestin of the adhesion protein desmoplakin in T47D breast cancer cells*.* *Breast Cancer Res*;**6:** R239-45.

16. Chidgey M Dawson C. (2007). Desmosomes: a role in cancer? *Br J Cancer*;**96:** 1783-7.

17. Hakimelahi S, Parker HR, Gilchrist AJ, Barry M, Li Z, Bleackley RC *et al.* (2000). Plakoglobin regulates the expression of the anti-apoptotic protein BCL-2*.* *J Biol Chem*;**275:** 10905-11.

18. Shiina H, Breault JE, Basset WW, Enokida H, Urakami S, Li LC *et al.* (2005). Functional Loss of the gamma-catenin gene through epigenetic and genetic pathways in human prostate cancer*.* *Cancer Res*;**65:** 2130-8.

19. Pyle-Chenault RA, Stolk JA, Molesh DA, Boyle-Harlan D, McNeill PD, Repasky EA *et al.* (2005). VSGP/F-spondin: a new ovarian cancer marker*.* *Tumour Biol*;**26:** 245-57.

20. Ross DT, Scherf U, Eisen MB, Perou CM, Rees C, Spellman P *et al.* (2000). Systematic variation in gene expression patterns in human cancer cell lines*.* *Nat Genet*;**24:** 227-35.

21. Simon I, Liu Y, Krall KL, Urban N, Wolfert RL, Kim NW *et al.* (2007). Evaluation of the novel serum markers B7-H4, Spondin 2, and DcR3 for diagnosis and early detection of ovarian cancer*.* *Gynecol Oncol*;**106:** 112-8.

22. Brueckl WM, Wirtz, R. M., Croner, R. S., Boxberger, F., Papadopoulos, T., Kirchner, T., Hahn, E. G., Hohenberger, W., Wein, A. (2007). Evaluation of Spondin-1 as a predictive marker for palliative 5-FU-based chemotherapy in metastatic colorectal cancer

*J Clin Oncol (Meeting Abstracts)* 4112.

23. King SJ Schroer TA. (2000). Dynactin increases the processivity of the cytoplasmic dynein motor*.* *Nat Cell Biol*;**2:** 20-4.

24. Bransfield KL, Askham JM, Leek JP, Robinson PAMighell AJ. (2006). Phenotypic changes associated with DYNACTIN-2 (DCTN2) over expression characterise SJSA-1 osteosarcoma cells*.* *Mol Carcinog*;**45:** 157-63.

25. Kang JaY, H. (2008). Targeting the Spindle Checkpoint in Cancer Chemotherapy (chapter from book "Checkpoint Responses in Cancer Therapy")*.* *Humana Press*

26. Bhat KM Setaluri V. (2007). Microtubule-associated proteins as targets in cancer chemotherapy*.* *Clin Cancer Res*;**13:** 2849-54.

27. Blackhall FH, Merry CL, Davies EJJayson GC. (2001). Heparan sulfate proteoglycans and cancer*.* *Br J Cancer*;**85:** 1094-8.

28. Davies EJ, Blackhall FH, Shanks JH, David G, McGown AT, Swindell R *et al.* (2004). Distribution and clinical significance of heparan sulfate proteoglycans in ovarian cancer*.* *Clin Cancer Res*;**10:** 5178-86.

29. Williams S, Ryan CJacobson C. (2008). Agrin and neuregulin, expanding roles and implications for therapeutics*.* *Biotechnol Adv*;**26:** 187-201.

30. Tatrai P, Dudas J, Batmunkh E, Mathe M, Zalatnai A, Schaff Z *et al.* (2006). Agrin, a novel basement membrane component in human and rat liver, accumulates in cirrhosis and hepatocellular carcinoma*.* *Lab Invest*;**86:** 1149-60.

31. Rascher G, Fischmann A, Kroger S, Duffner F, Grote EHWolburg H. (2002). Extracellular matrix and the blood-brain barrier in glioblastoma multiforme: spatial segregation of tenascin and agrin*.* *Acta Neuropathol*;**104:** 85-91.

32. Selva EM Perrimon N. (2001). Role of heparan sulfate proteoglycans in cell signaling and cancer*.* *Adv Cancer Res*;**83:** 67-80.

33. Presland RB, Kuechle MK, Lewis SP, Fleckman PDale BA. (2001). Regulated expression of human filaggrin in keratinocytes results in cytoskeletal disruption, loss of cell-cell adhesion, and cell cycle arrest*.* *Exp Cell Res*;**270:** 199-213.

34. Kuechle MK, Presland RB, Lewis SP, Fleckman PDale BA. (2000). Inducible expression of filaggrin increases keratinocyte susceptibility to apoptotic cell death*.* *Cell Death Differ*;**7:** 566-73.

35. Dale BA, Resing KALonsdale-Eccles JD. (1985). Filaggrin: a keratin filament associated protein*.* *Ann N Y Acad Sci*;**455:** 330-42.

36. Dale BA. (1985). Filaggrin, the matrix protein of keratin*.* *Am J Dermatopathol*;**7:** 65-8.

37. van der Watt PJ, Maske CP, Hendricks DT, Parker MI, Denny L, Govender D *et al.* (2009). The Karyopherin proteins, Crm1 and Karyopherin beta1, are overexpressed in cervical cancer and are critical for cancer cell survival and proliferation*.* *Int J Cancer*;**124:** 1829-40.

38. Seedorf M Silver PA. (1997). Importin/karyopherin protein family members required for mRNA export from the nucleus*.* *Proc Natl Acad Sci U S A*;**94:** 8590-5.

39. Gluz O, Wild P, Meiler R, Diallo-Danebrock R, Ting E, Mohrmann S *et al.* (2008). Nuclear karyopherin alpha2 expression predicts poor survival in patients with advanced breast cancer irrespective of treatment intensity*.* *Int J Cancer*;**123:** 1433-8.

40. Teng SC, Wu KJ, Tseng SF, Wong CWKao L. (2006). Importin KPNA2, NBS1, DNA repair and tumorigenesis*.* *J Mol Histol*;**37:** 293-9.

41. Vasiliou V Nebert DW. (2005). Analysis and update of the human aldehyde dehydrogenase (ALDH) gene family*.* *Hum Genomics*;**2:** 138-43.

42. Marchitti SA, Brocker C, Stagos DVasiliou V. (2008). Non-P450 aldehyde oxidizing enzymes: the aldehyde dehydrogenase superfamily*.* *Expert Opin Drug Metab Toxicol*;**4:** 697-720.

43. Di Michele M, Della Corte A, Cicchillitti L, Del Boccio P, Urbani A, Ferlini C *et al.* (2009). A proteomic approach to paclitaxel chemoresistance in ovarian cancer cell lines*.* *Biochim Biophys Acta*;**1794:** 225-36.

44. Murray GI, Paterson PJ, Weaver RJ, Ewen SW, Melvin WTBurke MD. (1993). The expression of cytochrome P-450, epoxide hydrolase, and glutathione S-transferase in hepatocellular carcinoma*.* *Cancer*;**71:** 36-43.

45. Theyer G, Schirmbock M, Thalhammer T, Sherwood ER, Baumgartner GHamilton G. (1993). Role of the MDR-1-encoded multiple drug resistance phenotype in prostate cancer cell lines*.* *J Urol*;**150:** 1544-7.

46. Murray GI, Taylor VE, McKay JA, Weaver RJ, Ewen SW, Melvin WT *et al.* (1995). The immunohistochemical localization of drug-metabolizing enzymes in prostate cancer*.* *J Pathol*;**177:** 147-52.

47. Authier F, Posner BIBergeron JJ. (1996). Insulin-degrading enzyme*.* *Clin Invest Med*;**19:** 149-60.

48. Yfanti C, Mengele K, Gkazepis A, Weirich G, Giersig C, Kuo WL *et al.* (2008). Expression of metalloprotease insulin-degrading enzyme insulysin in normal and malignant human tissues*.* *Int J Mol Med*;**22:** 421-31.

49. Weirich G, Mengele K, Yfanti C, Gkazepis A, Hellmann D, Welk A *et al.* (2008). Immunohistochemical evidence of ubiquitous distribution of the metalloendoprotease insulin-degrading enzyme (IDE; insulysin) in human non-malignant tissues and tumor cell lines*.* *Biol Chem*;**389:** 1441-5.

50. Radulescu RT, Hufnagel C, Luppa P, Hellebrand H, Kuo WL, Rosner MR *et al.* (2007). Immunohistochemical demonstration of the zinc metalloprotease insulin-degrading enzyme in normal and malignant human breast: correlation with tissue insulin levels*.* *Int J Oncol*;**30:** 73-80.

51. Yamazaki T, Sasaki N, Nishi M, Yamazaki D, Ikeda A, Okuno Y *et al.* (2007). Augmentation of drug-induced cell death by ER protein BRI3BP*.* *Biochem Biophys Res Commun*;**362:** 971-5.

52. Ron D Walter P. (2007). Signal integration in the endoplasmic reticulum unfolded protein response*.* *Nat Rev Mol Cell Biol*;**8:** 519-29.

53. Bertram J, Palfner K, Hiddemann WKneba M. (1998). Overexpression of ribosomal proteins L4 and L5 and the putative alternative elongation factor PTI-1 in the doxorubicin resistant human colon cancer cell line LoVoDxR*.* *Eur J Cancer*;**34:** 731-6.

54. Vaarala MH, Porvari KS, Kyllonen AP, Mustonen MV, Lukkarinen OVihko PT. (1998). Several genes encoding ribosomal proteins are over-expressed in prostate-cancer cell lines: confirmation of L7a and L37 over-expression in prostate-cancer tissue samples*.* *Int J Cancer*;**78:** 27-32.

55. Meric F Hunt KK. (2002). Translation initiation in cancer: a novel target for therapy*.* *Mol Cancer Ther*;**1:** 971-9.

56. Dong Z Zhang JT. (2006). Initiation factor eIF3 and regulation of mRNA translation, cell growth, and cancer*.* *Crit Rev Oncol Hematol*;**59:** 169-80.

57. Choy L Derynck R. (1998). The type II transforming growth factor (TGF)-beta receptor-interacting protein TRIP-1 acts as a modulator of the TGF-beta response*.* *J Biol Chem*;**273:** 31455-62.

58. Pelicano H, Xu RH, Du M, Feng L, Sasaki R, Carew JS *et al.* (2006). Mitochondrial respiration defects in cancer cells cause activation of Akt survival pathway through a redox-mediated mechanism*.* *J Cell Biol*;**175:** 913-23.

59. Carew JS Huang P. (2002). Mitochondrial defects in cancer*.* *Mol Cancer*;**1:** 9.

60. Board M, Humm SNewsholme EA. (1990). Maximum activities of key enzymes of glycolysis, glutaminolysis, pentose phosphate pathway and tricarboxylic acid cycle in normal, neoplastic and suppressed cells*.* *Biochem J*;**265:** 503-9.

61. Quaroni A Paul EC. (1999). Cytocentrin is a Ral-binding protein involved in the assembly and function of the mitotic apparatus*.* *J Cell Sci*;**112 ( Pt 5):** 707-18.

62. Lim KH, Baines AT, Fiordalisi JJ, Shipitsin M, Feig LA, Cox AD *et al.* (2005). Activation of RalA is critical for Ras-induced tumorigenesis of human cells*.* *Cancer Cell*;**7:** 533-45.

63. Chien Y, Kim S, Bumeister R, Loo YM, Kwon SW, Johnson CL *et al.* (2006). RalB GTPase-mediated activation of the IkappaB family kinase TBK1 couples innate immune signaling to tumor cell survival*.* *Cell*;**127:** 157-70.

64. Oxford G, Owens CR, Titus BJ, Foreman TL, Herlevsen MC, Smith SC *et al.* (2005). RalA and RalB: antagonistic relatives in cancer cell migration*.* *Cancer Res*;**65:** 7111-20.

65. Lim KH, O'Hayer K, Adam SJ, Kendall SD, Campbell PM, Der CJ *et al.* (2006). Divergent roles for RalA and RalB in malignant growth of human pancreatic carcinoma cells*.* *Curr Biol*;**16:** 2385-94.

66. Zhang Y, Parker WB, Sorscher EJEalick SE. (2005). PNP anticancer gene therapy*.* *Curr Top Med Chem*;**5:** 1259-74.

67. Satoh MS Lindahl T. (1992). Role of poly(ADP-ribose) formation in DNA repair*.* *Nature*;**356:** 356-8.

68. Tewari M, Quan LT, O'Rourke K, Desnoyers S, Zeng Z, Beidler DR *et al.* (1995). Yama/CPP32 beta, a mammalian homolog of CED-3, is a CrmA-inhibitable protease that cleaves the death substrate poly(ADP-ribose) polymerase*.* *Cell*;**81:** 801-9.

69. Trucco C, Oliver FJ, de Murcia GMenissier-de Murcia J. (1998). DNA repair defect in poly(ADP-ribose) polymerase-deficient cell lines*.* *Nucleic Acids Res*;**26:** 2644-9.

70. Oliver FJ, de la Rubia G, Rolli V, Ruiz-Ruiz MC, de Murcia GMurcia JM. (1998). Importance of poly(ADP-ribose) polymerase and its cleavage in apoptosis. Lesson from an uncleavable mutant*.* *J Biol Chem*;**273:** 33533-9.

71. Nicholson DW, Ali A, Thornberry NA, Vaillancourt JP, Ding CK, Gallant M *et al.* (1995). Identification and inhibition of the ICE/CED-3 protease necessary for mammalian apoptosis*.* *Nature*;**376:** 37-43.

72. Sakamoto-Hojo ET Balajee AS. (2008). Targeting poly (ADP) ribose polymerase I (PARP-1) and PARP-1 interacting proteins for cancer treatment*.* *Anticancer Agents Med Chem*;**8:** 402-16.

73. Peralta-Leal A, Rodriguez MIOliver FJ. (2008). Poly(ADP-ribose)polymerase-1 (PARP-1) in carcinogenesis: potential role of PARP inhibitors in cancer treatment*.* *Clin Transl Oncol*;**10:** 318-23.

74. Lord CJ Ashworth A. (2008). Targeted therapy for cancer using PARP inhibitors*.* *Curr Opin Pharmacol*;**8:** 363-9.

75. Helleday T, Bryant HESchultz N. (2005). Poly(ADP-ribose) polymerase (PARP-1) in homologous recombination and as a target for cancer therapy*.* *Cell Cycle*;**4:** 1176-8.

76. Chalmers AJ. (2009). The potential role and application of PARP inhibitors in cancer treatment*.* *Br Med Bull*;**89:** 23-40.

77. Bey EA, Bentle MS, Reinicke KE, Dong Y, Yang CR, Girard L *et al.* (2007). An NQO1- and PARP-1-mediated cell death pathway induced in non-small-cell lung cancer cells by beta-lapachone*.* *Proc Natl Acad Sci U S A*;**104:** 11832-7.

78. Rohe HJ, Ahmed IS, Twist KECraven RJ. (2009). PGRMC1 (progesterone receptor membrane component 1): a targetable protein with multiple functions in steroid signaling, P450 activation and drug binding*.* *Pharmacol Ther*;**121:** 14-9.

79. Losel RM, Besong D, Peluso JJWehling M. (2008). Progesterone receptor membrane component 1--many tasks for a versatile protein*.* *Steroids*;**73:** 929-34.

80. Peluso JJ, Liu X, Saunders MM, Claffey KPPhoenix K. (2008). Regulation of ovarian cancer cell viability and sensitivity to cisplatin by progesterone receptor membrane component-1*.* *J Clin Endocrinol Metab*;**93:** 1592-9.

81. Peluso JJ, Romak JLiu X. (2008). Progesterone receptor membrane component-1 (PGRMC1) is the mediator of progesterone's antiapoptotic action in spontaneously immortalized granulosa cells as revealed by PGRMC1 small interfering ribonucleic acid treatment and functional analysis of PGRMC1 mutations*.* *Endocrinology*;**149:** 534-43.

82. Cahill MA. (2007). Progesterone receptor membrane component 1: an integrative review*.* *J Steroid Biochem Mol Biol*;**105:** 16-36.

83. Craven RJ. (2008). PGRMC1: a new biomarker for the estrogen receptor in breast cancer*.* *Breast Cancer Res*;**10:** 113.

84. Escobar E, Rodriguez-Reyna TS, Arrieta OSotelo J. (2004). Angiotensin II, cell proliferation and angiogenesis regulator: biologic and therapeutic implications in cancer*.* *Curr Vasc Pharmacol*;**2:** 385-99.

85. Suganuma T, Ino K, Shibata K, Kajiyama H, Nagasaka T, Mizutani S *et al.* (2005). Functional expression of the angiotensin II type 1 receptor in human ovarian carcinoma cells and its blockade therapy resulting in suppression of tumor invasion, angiogenesis, and peritoneal dissemination*.* *Clin Cancer Res*;**11:** 2686-94.

86. Herr D, Rodewald M, Fraser HM, Hack G, Konrad R, Kreienberg R *et al.* (2008). Potential role of Renin-Angiotensin-system for tumor angiogenesis in receptor negative breast cancer*.* *Gynecol Oncol*;**109:** 418-25.

87. Marzesco AM, Dunia I, Pandjaitan R, Recouvreur M, Dauzonne D, Benedetti EL *et al.* (2002). The small GTPase Rab13 regulates assembly of functional tight junctions in epithelial cells*.* *Mol Biol Cell*;**13:** 1819-31.

88. Stenmark H Olkkonen VM. (2001). The Rab GTPase family*.* *Genome Biol*;**2:** REVIEWS3007.

89. Chou TC. (2006). Theoretical basis, experimental design, and computerized simulation of synergism and antagonism in drug combination studies*.* *Pharmacol Rev*;**58:** 621-81.
