# Supplementary material for: Purine Nucleoside Phosphorylase mediated molecular chemotherapy and conventional chemotherapy: A tangible union against chemoresistant cancer
Source: BMC Cancer. 2011 Aug 24;11:368. doi: 10.1186/1471-2407-11-368 (PMC3185280; doi:10.1186/1471-2407-11-368)
Supplement: Additional file 6 — Table S5. Docetaxel (nM) needed to kill 50% of ovarian cancer cells (IC50). [file 1471-2407-11-368-S6.DOC]

**Additional File 6**

**Title: Table S5**

**Description: Docetaxel (nM) needed to kill 50% of ovarian cancer cells (IC_50_)**

**Table S5 Docetaxel (nM) needed to kill 50% of ovarian cancer cells (IC_50_)**

| **Ovarian Cancer**  **Cell Line** | **IC_50_ ±SEM (nM)^1^** | | | |
| --- | --- | --- | --- | --- |
|  | **Day 2**  **(R^2^ )^3^** | **Day 3**  **(R^2^ )** | **Day 4**  **(R^2^ )** | **Day 5^2^**  **(R^2^ )** |
| **SKOV-3** | 1.459±1.1  (0.96) | 0.498±0.7  (0.93) | 0.375±0.6  (0.970 | 0.310±0.7  (0.96) |
| **OVCAR-3** | 2.901±1.7  (0.94) | 1.255±1.7  (0.98) | 1.020±1.1  (0.98) | 0.614±1.1  (0.99) |
| **Caov-3** | 3.511±2.1  (0.92) | 1.494±1.9  (0.98) | 1.467±1.6  (0.97) | 1.212±1.4  (0.96) |
| **A-2780** | 0.685±0.7  (0.97) | 0.479±1.1  (0.96) | 0.186±0.4  (0.96) | 0.066±0.3^4^  (0.97) |

^1^Calculated from dose response curves shown in figure s4

^2^ The IC_50_ values obtained after 5days of drug treatment were used in drug combination studies

^3^ R^2^ values >0.9 suggest that data is reliable and fits the statistical considerations

^4^Note: The IC50 value in A-2780 cells was lower than the lowest dose tested.
