# Supplementary material for: Purine Nucleoside Phosphorylase mediated molecular chemotherapy and conventional chemotherapy: A tangible union against chemoresistant cancer
Source: BMC Cancer. 2011 Aug 24;11:368. doi: 10.1186/1471-2407-11-368 (PMC3185280; doi:10.1186/1471-2407-11-368)
Supplement: Additional file 8 — Table S6. Dose Reduction Index (DRI) values for different modalities when used in combination in platinum resistant ovarian cancer cells. [file 1471-2407-11-368-S8.DOC]

**Additional File 8**

**Title: Table S6**

**Description: : Dose Reduction Index (DRI) values for different modalities when used in combination in platinum resistant ovarian cancer cells.**

| **SKOV-3** | | | | | | | | | | | |  | | **OVCAR-3** | | | | | | | | | | | | |
| --- | --- | --- | --- | --- | --- | --- | --- | --- | --- | --- | --- | --- | --- | --- | --- | --- | --- | --- | --- | --- | --- | --- | --- | --- | --- | --- |
| **Fraction affected** | **Drug Alone**  **(xIC_50_)** | | | | | | **Dose Reduction Index (xIC_50_)** | | | | |  | | **Fraction affected** | **Drug Alone**  **(xIC_50_)** | | | | | | **Dose Reduction Index (xIC_50_)** | | | | | |
| ***(Fa)*** | ***Docetaxel*** | | | ***Carboplatin*** | | | ***Docetaxel*** | | ***Carboplatin*** | | |  | | ***(Fa)*** | ***Docetaxel*** | | | ***Carboplatin*** | | | ***Docetaxel*** | | ***Carboplatin*** | | | |
| *0.10* | 0.31 | | | 0.14 | | | 1.87 | | 1.45 | | |  | | *0.10* | 0.22 | | | 0.28 | | | 1.54 | | 1.73 | | | |
| *0.25* | 1.25 | | | 0.43 | | | 2.25 | | 1.56 | | |  | | *0.25* | 0.79 | | | 0.85 | | | 1.79 | | 2.05 | | | |
| *0.50* | 5.01 | | | 1.34 | | | 2.70 | | 1.68 | | |  | | *0.50* | 2.76 | | | 2.16 | | | 2.06 | | 2.44 | | | |
| *0.75* | 20.16 | | | 4.18 | | | 3.24 | | 1.81 | | |  | | *0.75* | 9.70 | | | 8.99 | | | 2.39 | | 2.89 | | | |
| *0.90* | 81.09 | | | 12.98 | | | 3.89 | | 1.95 | | |  | | *0.90* | 34.07 | | | 31.34 | | | 2.76 | | 3.44 | | | |
| ***(Fa)*** | ***GDEPT*** | | | | ***Carboplatin*** | | ***GDEPT*** | | | ***Carboplatin*** | |  | | ***(Fa)*** | ***GDEPT*** | | | | ***Carboplatin*** | | ***GDEPT*** | | | | ***Carboplatin*** | |
| *0.10* | 0.44 | | | | 0.14 | | 5.21 | | | 2.84 | |  | | *0.10* | 0.17 | | | | 0.28 | | 2.79 | | | | 3.99 | |
| *0.25* | 1.91 | | | | 0.43 | | 8.02 | | | 3.65 | |  | | *0.25* | 0.60 | | | | 0.85 | | 3.97 | | | | 6.02 | |
| *0.50* | 8.22 | | | | 1.34 | | 12.36 | | | 4.70 | |  | | *0.50* | 2.03 | | | | 2.16 | | 5.66 | | | | 9.08 | |
| *0.75* | 35.46 | | | | 4.18 | | 19.03 | | | 6.05 | |  | | *0.75* | 6.93 | | | | 8.99 | | 8.08 | | | | 13.70 | |
| *0.90* | 152.91 | | | | 12.98 | | 29.30 | | | 7.78 | |  | | *0.90* | 23.64 | | | | 31.34 | | 11.51 | | | | 20.68 | |
| ***(Fa)*** | ***GDEPT*** | ***Doc*** | | | | ***Car*** | ***GDEPT*** | | ***Doc*** | | ***Car*** |  | | ***(Fa)*** | ***GDEPT*** | ***Doc*** | | | | ***Car*** | ***GDEPT*** | ***Doc*** | | | | ***Car*** |
| *0.10* | 0.44 | 0.31 | | | | 0.14 | 5.76 | | 4.04 | | 3.14 |  |  | *0.10* | 0.17 | 0.22 | | | | 0.28 | 12.58 | 16.12 | | | | 18.00 |
| *0.25* | 1.91 | 1.25 | | | | 0.43 | 8.97 | | 5.86 | | 4.08 |  | | *0.25* | 0.60 | 0.79 | | | | 0.85 | 15.82 | 20.87 | | | | 23.96 |
| *0.50* | 8.22 | 5.01 | | | | 1.34 | 13.96 | | 8.51 | | 5.31 |  | | *0.50* | 2.03 | 2.76 | | | | 2.16 | 19.89 | 27.02 | | | | 31.87 |
| *0.75* | 35.46 | 20.16 | | | | 4.18 | 21.73 | | 12.36 | | 6.91 |  | | *0.75* | 6.93 | 9.70 | | | | 8.99 | 25.00 | 34.98 | | | | 42.41 |
| *0.90* | 152.91 | 81.09 | | | | 12.98 | 33.83 | | 17.94 | | 8.99 |  | | *0.90* | 23.64 | 34.07 | | | | 31.34 | 31.43 | 45.29 | | | | 56.43 |
| ***(Fa)*** | ***GDEPT*** | | ***Docetaxel*** | | | | ***GDEPT*** | ***Docetaxel*** | | | | |  | ***(Fa)*** | ***GDEPT*** | | ***Docetaxel*** | | | | ***GDEPT*** | | | ***Docetaxel*** | | |
| *0.10* | 0.44 | | 0.31 | | | | 3.18 | 2.23 | | | | |  | *0.10* | 0.17 | | 0.22 | | | | 3.24 | | | 4.15 | | |
| *0.25* | 1.91 | | 1.25 | | | | 4.41 | 2.88 | | | | |  | *0.25* | 0.60 | | 0.79 | | | | 5.08 | | | 6.70 | | |
| *0.50* | 8.22 | | 5.01 | | | | 6.12 | 3.73 | | | | |  | *0.50* | 2.03 | | 2.76 | | | | 7.97 | | | 10.82 | | |
| *0.75* | 35.46 | | 20.16 | | | | 8.49 | 4.83 | | | | |  | *0.75* | 6.93 | | 9.70 | | | | 12.49 | | | 17.48 | | |
| *0.90* | 152.91 | | 81.09 | | | | 11.78 | 6.25 | | | | |  | *0.90* | 23.64 | | 34.07 | | | | 19.59 | | | 28.23 | | |

**Table S6: Dose Reduction Index (DRI) values for different modalities when used in combination in platinum resistant ovarian cancer cells.**
