# Supplementary material for: Purine Nucleoside Phosphorylase mediated molecular chemotherapy and conventional chemotherapy: A tangible union against chemoresistant cancer
Source: BMC Cancer. 2011 Aug 24;11:368. doi: 10.1186/1471-2407-11-368 (PMC3185280; doi:10.1186/1471-2407-11-368)
Supplement: Additional file 9 — Table S7. Interactions between different components of the combination treatments at different drug combination ratios in OC cells. [file 1471-2407-11-368-S9.DOC]

**Additional File 9**

**Title: Table S7**

**Description: Interactions between different components of the combination treatments at different drug combination ratios in OC cells**

**Table S7: Interactions between different components of the combination treatments at different drug combination ratios** in OC cells

| **Modalities** | **Cell Line** | **Combination Index (CI value)**  **To achieve 50% cell growth inhibition** | | | **R^2^** | | **Interaction** |
| --- | --- | --- | --- | --- | --- | --- | --- |
|  |  | **Ratio of Drugs (at IC_50_)** | | |  |  |  |
|  |  | **1:2** | **1:4** | **2:1** |  |  |  |
| **Docetaxel + Carboplatin** | SKOV-3 | 1.11 | 1.11 | 0.96 | 0.97 | Additive  **+** | |
|  | OVCAR-3 | 0. 94 | 0.96 | 0.89 | 0.98 | Additive  **+** | |
| **GDEPT+ Docetaxel** | SKOV-3 | 0.52 | 0.43 | 0.62 | 0.96 | Synergistic  **+++** | |
|  | OVCAR-3 | 0.22 | 0.21 | 0.16 | 0.97 | Strong Synergy  **++++** | |
| **GDEPT+ Carboplatin** | SKOV-3 | 0.30 | 0.29 | 0.33 | 0.98 | Strong Synergy  **++++** | |
|  | OVCAR-3 | 0.24 | 0.29 | 0.21 | 0.97 | Strong Synergy  ++++ | |
| **GDEPT + Docetaxel + Carboplatin** | SKOV-3 | 0.41 | 0.45 | 0.37 | 0.95 | Synergistic  **+++** | |
|  | OVCAR-3 | 0.09 | 0.07 | 0.02 | 0.96 | Very Strong Synergy  **+++++** | |
