# Supplementary material for: Purine Nucleoside Phosphorylase mediated molecular chemotherapy and conventional chemotherapy: A tangible union against chemoresistant cancer
Source: BMC Cancer. 2011 Aug 24;11:368. doi: 10.1186/1471-2407-11-368 (PMC3185280; doi:10.1186/1471-2407-11-368)
Supplement: Additional file 10 — Table S8. Genes/Proteins expressions and their correlation with OC outcomes. [file 1471-2407-11-368-S10.DOC]

| Family **Table S8: Genes/Proteins expressions and their correlation with OC outcomes.**  **Additional File 10**  **Title: Table S8**  **Description: Genes/Proteins expressions and their correlation with OC outcomes.** | Member Protein | Role in OC progression/treatment* | **Ref.** |
| --- | --- | --- | --- |
| Genes/Proteins whose down regulation is a favourable outcome for OC | | | |
| **Anti-apoptotic members of B-Cell CLL/Lymphoma-**2 (**BCL-2) family** | BCL-2 | Anti apoptotic; promotes cell survival in response to apoptotic stimuli through inhibition of mitochondrial cytochrome c release; frequently overexpressed in OC; Inhibits drug-induced apoptosis; a prognostic marker and predictor of chemosensitivity | [1-12] |
| **Inhibitor of Apoptosis (IAP)** | **Survivin** | Anti apoptotic; inhibition of apoptosis and promotion of cell division by binding and inhibiting Caspase-3 in G2/M phase of cell cycle; frequently overexpressed in different histological types of OC; a prognostic marker and predictor of chemosensitivity | [13-18] |
| Genes/Proteins whose up regulation is a favourable outcome for OC | | | |
| **BCL-2**  **Pro-apoptosis** | **BAX** | Pro-apoptotic; tumor suppressor; results apoptosis in both p53 dependent and independent pathways; BAX sensitises ovarian cancer cell lines to paclitaxel *in vitro;* a predictor of responsiveness to paclitaxel or platinum based chemotherapy in OC patients | [1, 5, 19-24] |
|  | BCL-2**-interacting** killer **(Bik)** | A proapoptotic gene often named as Nbk**/**bik (Natural Born Killer)**;** contains only one of the BCL-2 homology regions, the BH3^3^ domain; forms heterodimers with various antiapoptotic proteins, such as BCL-2 and Bcl-X_L_, to inhibit their antiapoptotic function; tumor cell sensitiser to chemotherapy e.g, doxorubicin | [25-29] |
|  | **BCL-2-related ovarian killer (Bok)** | Proapoptotic; absence of BH4 domain, which is found only in anti-apoptotic BCL-2 proteins; heterodimerizes only with selective anti-apoptotic BCL-2 proteins; does not bind to BCL-2 *(increased expression of BCL-2 does not affect its proapoptotic nature)*; a cell cycle regulator, which sensitises cells to stress induced apoptosis e.g., *Flavopiridol* | [30-32] |
| **Caspases** | **Caspase-7** | The ‘executioner’ caspase**-**7; activation leads to apoptosis; activated in OC cells in response to a variety of chemotherapeutic or biologic agents; may help to evaluate the patient’s responsiveness to chemotherapy | [33-36] |
|  | **Caspase-9** | The ‘initiator’ caspase**-**9; activation leads to apoptosis; activated in OC cells in response to a variety of chemotherapeutic or biologic agents; may help to evaluate the patient’s responsiveness to chemotherapy | [35-37] |

**References:**

1. I. Ziolkowska-Seta, R. Madry, E. Kraszewska, T. Szymanska, A. Timorek, A. RembiszewskaJ. Kupryjanczyk, TP53, BCL-2 and BAX analysis in 199 ovarian cancer patients treated with taxane-platinum regimens, Gynecol Oncol 112 (2009) 179-84

2. F. Tas, D. Duranyildiz, H. Oguz, H. Camlica, V. YasaseverE. Topuz, The value of serum bcl-2 levels in advanced epithelial ovarian cancer, Med Oncol 23 (2006) 213-7

3. R. A. Sagarra, L. A. Andrade, E. Z. Martinez, G. A. Pinto, K. J. SyrjanenS. F. Derchain, P53 and Bcl-2 as prognostic predictors in epithelial ovarian cancer, Int J Gynecol Cancer 12 (2002) 720-7

4. A. Protopapas, E. Diakomanolis, A. Bamias, S. Milingos, S. Markaki, C. Papadimitriou, A. M. DimopoulosS. Michalas, The prognostic significance of the immunohistochemical expression of p53, bcl-2, c-erb B-2 and cathepsin-D in ovarian cancer patients receiving platinum with cyclophosphamide or paclitaxel chemotherapy, Eur J Gynaecol Oncol 25 (2004) 225-9

5. D. Marx, C. Binder, H. Meden, T. Lenthe, T. Ziemek, T. Hiddemann, W. KuhnA. Schauer, Differential expression of apoptosis associated genes bax and bcl-2 in ovarian cancer, Anticancer Res 17 (1997) 2233-40

6. Y. Mano, Y. Kikuchi, K. Yamamoto, T. Kita, J. Hirata, T. Tode, K. IshiiI. Nagata, Bcl-2 as a predictor of chemosensitivity and prognosis in primary epithelial ovarian cancer, Eur J Cancer 35 (1999) 1214-9

7. V. Malamou-Mitsi, O. Crikoni, E. Timotheadou, G. Aravantinos, E. Vrettou, N. AgnantisG. Fountzilas, Prognostic significance of HER-2, p53 and Bcl-2 in patients with epithelial ovarian cancer, Anticancer Res 27 (2007) 1157-65

8. S. K. Kassim, H. S. Ali, M. M. Sallam, S. T. Fayed, L. S. Seada, E. abd-Elkawy, M. A. SeadaA. Khalifa, Increased bcl-2 expression is associated with primary resistance to chemotherapy in human epithelial ovarian cancer, Clin Biochem 32 (1999) 333-8

9. H. Camlica, D. Duranyildiz, F. TasV. Yasasever, Statistical interpretation of CA125 and Bcl-2 in serum of patients with late stage ovarian cancer, Am J Clin Oncol 31 (2008) 585-8

10. N. S. Anderson, Y. Bermudez, D. Badgwell, R. Chen, S. V. Nicosia, R. C. Bast, Jr.P. A. Kruk, Urinary levels of Bcl-2 are elevated in ovarian cancer patients, Gynecol Oncol 112 (2009) 60-7

11. K. W. Yip J. C. Reed, Bcl-2 family proteins and cancer, Oncogene 27 (2008) 6398-406

12. Z. Humlova, Protooncogene bcl-2 in process of apoptosis. Review article, Sb Lek 103 (2002) 419-25

13. P. Athanassiadou, D. Grapsa, P. Athanassiades, M. Gonidi, A. M. Athanassiadou, A. TsipisE. Patsouris, The prognostic significance of COX-2 and survivin expression in ovarian cancer, Pathol Res Pract 204 (2008) 241-9

14. Z. Liguang, L. Peishu, M. Hongluan, J. Hong, W. Rong, M. S. WachtelE. E. Frezza, Survivin expression in ovarian cancer, Exp Oncol 29 (2007) 121-5

15. B. Zhang, J. S. Pan, J. Y. Liu, S. P. Han, G. HuB. Wang, Effects of chemotherapy and/or radiotherapy on survivin expression in ovarian cancer, Methods Find Exp Clin Pharmacol 28 (2006) 619-25

16. Z. Wang, Y. XieH. Wang, Changes in survivin messenger RNA level during chemotherapy treatment in ovarian cancer cells, Cancer Biol Ther 4 (2005) 716-9

17. G. Ferrandina, F. Legge, E. Martinelli, F. O. Ranelletti, G. F. Zannoni, L. Lauriola, M. Gessi, V. GallottaG. Scambia, Survivin expression in ovarian cancer and its correlation with clinico-pathological, surgical and apoptosis-related parameters, Br J Cancer 92 (2005) 271-7

18. N. Zaffaroni, M. Pennati, G. Colella, P. Perego, R. Supino, L. Gatti, S. Pilotti, F. ZuninoM. G. Daidone, Expression of the anti-apoptotic gene survivin correlates with taxol resistance in human ovarian cancer, Cell Mol Life Sci 59 (2002) 1406-12

19. M. Schuyer, M. E. van der Burg, S. C. Henzen-Logmans, J. H. Fieret, J. G. Klijn, M. P. Look, J. A. Foekens, G. StoterE. M. Berns, Reduced expression of BAX is associated with poor prognosis in patients with epithelial ovarian cancer: a multifactorial analysis of TP53, p21, BAX and BCL-2, Br J Cancer 85 (2001) 1359-67

20. Y. Tsuruta, M. Mandai, I. Konishi, H. Kuroda, T. Kusakari, Y. Yura, A. A. Hamid, I. Tamura, M. KariyaS. Fujii, Combination effect of adenovirus-mediated pro-apoptotic bax gene transfer with cisplatin or paclitaxel treatment in ovarian cancer cell lines, Eur J Cancer 37 (2001) 531-41

21. W. O. Arafat, J. Gomez-Navarro, J. Xiang, M. N. Barnes, P. Mahasreshti, R. D. Alvarez, G. P. Siegal, A. O. Badib, D. Buchsbaum, D. T. CurielM. A. Stackhouse, An adenovirus encoding proapoptotic Bax induces apoptosis and enhances the radiation effect in human ovarian cancer, Mol Ther 1 (2000) 545-54

22. J. Xiang, J. Gomez-Navarro, W. Arafat, B. Liu, S. D. Barker, R. D. Alvarez, G. P. SiegalD. T. Curiel, Pro-apoptotic treatment with an adenovirus encoding Bax enhances the effect of chemotherapy in ovarian cancer, J Gene Med 2 (2000) 97-106

23. Y. T. Tai, T. Strobel, D. KufeS. A. Cannistra, In vivo cytotoxicity of ovarian cancer cells through tumor-selective expression of the BAX gene, Cancer Res 59 (1999) 2121-6

24. Y. T. Tai, S. Lee, E. Niloff, C. Weisman, T. StrobelS. A. Cannistra, BAX protein expression and clinical outcome in epithelial ovarian cancer, J Clin Oncol 16 (1998) 2583-90

25. Y. Zou, H. Peng, B. Zhou, Y. Wen, S. C. Wang, E. M. TsaiM. C. Hung, Systemic tumor suppression by the proapoptotic gene bik, Cancer Res 62 (2002) 8-12

26. Y. Tong, Q. Yang, C. Vater, L. K. Venkatesh, D. Custeau, T. Chittenden, G. ChinnaduraiH. Gourdeau, The pro-apoptotic protein, Bik, exhibits potent antitumor activity that is dependent on its BH3 domain, Mol Cancer Ther 1 (2001) 95-102

27. M. Nikrad, T. Johnson, H. Puthalalath, L. Coultas, J. AdamsA. S. Kraft, The proteasome inhibitor bortezomib sensitizes cells to killing by death receptor ligand TRAIL via BH3-only proteins Bik and Bim, Mol Cancer Ther 4 (2005) 443-9

28. J. Han, P. SabbatiniE. White, Induction of apoptosis by human Nbk/Bik, a BH3-containing protein that interacts with E1B 19K, Mol Cell Biol 16 (1996) 5857-64

29. J. M. Boyd, G. J. Gallo, B. Elangovan, A. B. Houghton, S. Malstrom, B. J. Avery, R. G. Ebb, T. Subramanian, T. Chittenden, R. J. Lutzet al., Bik, a novel death-inducing protein shares a distinct sequence motif with Bcl-2 family proteins and interacts with viral and cellular survival-promoting proteins, Oncogene 11 (1995) 1921-8

30. S. Y. Hsu, A. Kaipia, E. McGee, M. LomeliA. J. Hsueh, Bok is a pro-apoptotic Bcl-2 protein with restricted expression in reproductive tissues and heterodimerizes with selective anti-apoptotic Bcl-2 family members, Proc Natl Acad Sci U S A 94 (1997) 12401-6

31. S. Y. Hsu A. J. Hsueh, A splicing variant of the Bcl-2 member Bok with a truncated BH3 domain induces apoptosis but does not dimerize with antiapoptotic Bcl-2 proteins in vitro, J Biol Chem 273 (1998) 30139-46

32. J. M. Rodriguez, M. A. Glozak, Y. MaW. D. Cress, Bok, Bcl-2-related Ovarian Killer, Is Cell Cycle-regulated and Sensitizes to Stress-induced Apoptosis, J Biol Chem 281 (2006) 22729-35

33. J. J. Bao, X. F. Le, R. Y. Wang, J. Yuan, L. Wang, E. N. Atkinson, R. LaPushin, M. Andreeff, B. Fang, Y. YuR. C. Bast, Jr., Reexpression of the tumor suppressor gene ARHI induces apoptosis in ovarian and breast cancer cells through a caspase-independent calpain-dependent pathway, Cancer Res 62 (2002) 7264-72

34. A. B. Alvero, M. K. MontagnaG. Mor, Correlation of caspase activity and in vitro chemo-response in epithelial ovarian cancer cell lines, Methods Mol Biol 414 (2008) 79-82

35. S. J. Riedl Y. Shi, Molecular mechanisms of caspase regulation during apoptosis, Nat Rev Mol Cell Biol 5 (2004) 897-907

36. A. Degterev, M. BoyceJ. Yuan, A decade of caspases, Oncogene 22 (2003) 8543-67

37. G. S. Wu Z. Ding, Caspase 9 is required for p53-dependent apoptosis and chemosensitivity in a human ovarian cancer cell line, Oncogene 21 (2002) 1-8
