# Supplementary material for: Purine Nucleoside Phosphorylase mediated molecular chemotherapy and conventional chemotherapy: A tangible union against chemoresistant cancer
Source: BMC Cancer. 2011 Aug 24;11:368. doi: 10.1186/1471-2407-11-368 (PMC3185280; doi:10.1186/1471-2407-11-368)
Supplement: Additional file 11 — Table S9. Summary of treatment related effects on different pro and anti-apoptotic proteins. [file 1471-2407-11-368-S11.DOC]

**Table S9:** **Summary of treatment related effects on different pro and anti-apoptotic proteins.**

**Additional File 11**

**Title: Table S9**

**Description: Summary of treatment related effects on different pro and anti-apoptotic proteins**

| **Protein** | **Potential role** | **Relative levels of proteins (up or down regulation in response to different treatments*** | | | | | | | |
| --- | --- | --- | --- | --- | --- | --- | --- | --- | --- |
|  |  | U^1^ | C^2^ | T^3^ | CT^4^ | G^5^ | GC^6^ | GT^7^ | GCT^8^ |
| **BCL-2** | Anti-apoptotic | - | - | **-** | - | **↓** | **↓↓** | **↓↓** | **↓↓↓** |
| **Survivin** | Belongs to Inhibitor of Apoptosis (IAP) family | - | - | - | **↓↓** | **↓↓** | **-** | **-** | **↓↓↓** |
| **BAX** | Pro-apoptotic; helps to release cytochrome c from mitochondria and also results in the activation of caspase-9 | - | - | **↑** | **-** | **↑** | **↑↑↑** | **↑↑** | **↑↑↑** |
| **BCL-2-Interacting Killer (Bik)** | Pro-apoptotic; able to bind to and antagonize anti-apoptotic BCL-2 family members including BCL-2, Bcl-xL | - | **↑** | **↑** | **↑↑** | **↑** | **↑↑** | **↑↑** | **↑↑↑** |
| **BCL-2 related Ovarian Killer (Bok)** | Pro-apoptotic; promotes both caspase-dependent and caspase-independent apoptosis | - | **↑** | **↑** | **-** | **-** | **↑** | **↑↑** | **↑↑** |
| **Pro- Caspase-7** | - | - | - | - | **-** | - | - | - | **-** |
| **Cleaved caspase-7** | Pro-apoptotic; ‘executioner’ caspase | - | **↑** | **↑** | **↑↑** | **↑↑** | **↑↑** | **↑↑** | **↑↑↑** |
| **Pro- Caspase-9** | - | - | - | - | **-** | - | - | - | **-** |
| **Cleaved Caspase-9** | Pro-apoptotic; ‘initiator’ caspase | - | - | - | **↑** | **↑** | **↑** | **↑** | **↑↑** |
| **PARP** | DNA repair in response to some form of stress | - | - | - | **-** | **↑** | **↑** | **↑** | **↑↑** |
| **Cleaved PARP** | Final stage of apoptosis and a marker for cells undergoing apoptosis | - | - | - | **↑** | **↑** | **↑↑** | **↑↑** | **↑↑↑** |

U^1^, Untreated; C^2^, Carboplatin; T^3^, Docetaxel; CT^4^, Carboplatin + Docetaxel; G^5^, PNP-GDEPT; GC^6^, PNP-GDEPT + Carboplatin; GT^7^, PNP-GDEPT + Docetaxel; GTC^8^, PNP-GDEPT + Carboplatin + Docetaxel

***Symbols used:** Un-effected -; Up regulated ↑; moderately up regulated ↑↑; strongly up regulated ↑↑↑; Down regulated ↓; moderately down regulated ↓↓; strongly down regulated ↓↓↓
